# Multi-Variable Multi-Metric Optimization of Self-Assembled Photocatalytic CO_2_ Reduction Performance Using Machine Learning Algorithms

**DOI:** 10.1021/jacs.4c01305

**Published:** 2024-05-20

**Authors:** Shannon A. Bonke, Giovanni Trezza, Luca Bergamasco, Hongwei Song, Santiago Rodríguez-Jiménez, Leif Hammarström, Eliodoro Chiavazzo, Erwin Reisner

**Affiliations:** † Yusuf Hamied Department of Chemistry, University of Cambridge, Lensfield Road, Cambridge CB2 1EW, United Kingdom; ‡ Department of Energy, Politecnico di Torino, Corso Duca degli Abruzzi 24, Turin 10129, Italy; § Department of Chemistry, Ångström Laboratory, Uppsala University, Box 523, Uppsala 75120, Sweden

## Abstract

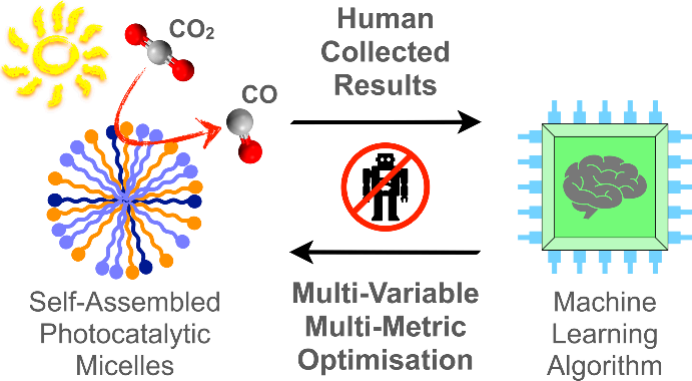

The sunlight-driven reduction of CO_2_ into fuels and platform chemicals is a promising approach to enable a circular economy. However, established optimization approaches are poorly suited to multivariable multimetric photocatalytic systems because they aim to optimize one performance metric while sacrificing the others and thereby limit overall system performance. Herein, we address this multimetric challenge by defining a metric for holistic system performance that takes multiple figures of merit into account, and employ a machine learning algorithm to efficiently guide our experiments through the large parameter matrix to make holistic optimization accessible for human experimentalists. As a test platform, we employ a five-component system that self-assembles into photocatalytic micelles for CO_2_-to-CO reduction, which we experimentally optimized to simultaneously improve yield, quantum yield, turnover number, and frequency while maintaining high selectivity. Leveraging the data set with machine learning algorithms allows quantification of each parameter’s effect on overall system performance. The buffer concentration is unexpectedly revealed as the dominating parameter for optimal photocatalytic activity, and is nearly four times more important than the catalyst concentration. The expanded use and standardization of this methodology to define and optimize holistic performance will accelerate progress in different areas of catalysis by providing unprecedented insights into performance bottlenecks, enhancing comparability, and taking results beyond comparison of subjective figures of merit.

## Introduction

Catalysis relies on a complex interplay of interdependent variables that must be optimized to meet a set of performance metrics. The challenge is exemplified by multicomponent photocatalytic systems where the parameter space is increasingly difficult to navigate due to the increasing number of variables required to provide supramolecular control (e.g., concentrations of reagents, additives, experimental variables). The optimization target is also unclear as there is limited standardization and a multitude of metrics to optimize (e.g., yield, quantum yield, selectivity, turnover number and frequency).^1,2^ Crucially, established and intuitive heuristic/human optimization approaches can only maximize 1 or 2 performance metrics simultaneously. This situation has led to selective optimizations where some metrics are prioritized in ways that have limited meaning to overall system performance because they use conditions that sacrifice other metrics; such as using a very low catalyst loading to reach a high turnover frequency (TOF) but having negligible product yield.^1^ The fundamental problem is that optimization to improve all figures of merit (holistic optimization) is not feasible with established protocols. The parameter space is too large to evaluate, the interactions of variables that affect multiple metrics are too complex, and no holistic figure of merit has been defined.^1,2^ New strategies are required to navigate the large parameter space and extract deeper understanding into how multiple variables interact and affect each metric to control overall system performance.^3−5^

Holistic optimization first requires all figures of merit to be connected via a mathematical description (an *objective function*) that evaluates to a single scalar value representing overall system performance. Iteratively varying parameters changes the value of each figure of merit, and thereby enables the objective function to quantify the change in overall performance. However, a complex system has too many combinatorial possibilities to test them all manually; for example, testing 10 concentrations of just 5 components gives 10^5^ possibilities. Finding the maximum in such a large parameter matrix could be addressed with “brute force” high-throughput approaches to rapidly screen all possibilities until a positive result is found.^6−9^ Alternatively, a learning algorithm can use Bayesian optimization to decipher how each variable affects overall system performance and vastly reduce the number of experiments required to maximize the objective function.^3,4,10−12^ Because learning algorithms change multiple parameters simultaneously and capture the relationships between variables,^3,4,10−12^ they can reduce the number of experiments required to optimize a complex system into range for human experimentalists. Thereby, use of learning algorithms enables all laboratories to holistically optimize their systems without expensive robotic systems. When robotic systems become more commonplace in the future, they will be guided by learning algorithms to further enhance the rate of scientific discovery.^5,13^

Supramolecular assembly is a frontier in molecular photocatalysis and biomimicry as it can accelerate charge transfer processes and eliminate diffusion limitations by providing optimal environments for reactions, in particular photocatalytic CO_2_ reduction.^14,15^ While judicial design of molecular components can enable self-assembly such as through amphiphilicity as used herein,^7,16,14,15^ the dependence of assembly on the components and environment results in a wide parameter space to explore and provides ideal systems to demonstrate holistic optimization of complex systems.

Herein we employ Bayesian optimization to steer an experimental campaign toward the simultaneous improvement of all key metrics for a five-component self-assembled photocatalytic molecular CO_2_ reduction system. This system was selected because the interdependent variables that control supramolecular structure make it challenging to optimize and representative of complex photocatalytic systems. Defining an objective function allowed simultaneous improvement of performance metrics, namely catalyst turnover number, turnover frequency, quantum yield and moles of product, without loss of selectivity. Concurrently, the use of Bayesian optimization reduced the number of experiments required to optimize the system from a theoretical 10^5^ down to ∼100 and made the work accessible for a human experimentalist with standard laboratory equipment. Acquiring a systematic data set that maps the parameter space then enabled the application of machine learning algorithms to extract relationships between the dependent and the independent variables. Furthermore, the role and importance of different parameters on the overall performance was revealed using Shapley additive explanations (SHAP).^17−19^ Finally, the problem dimensionality was reduced and parameter regions likely to further enhance system performance are exposed by controlling feature grouping.^20^ Thereby, the system was holistically optimized and deeper insight into the ruling parameters for each figure of merit was extracted.

## Results and Discussion

### Assembly of photocatalytic micelles

**Figure 1:**
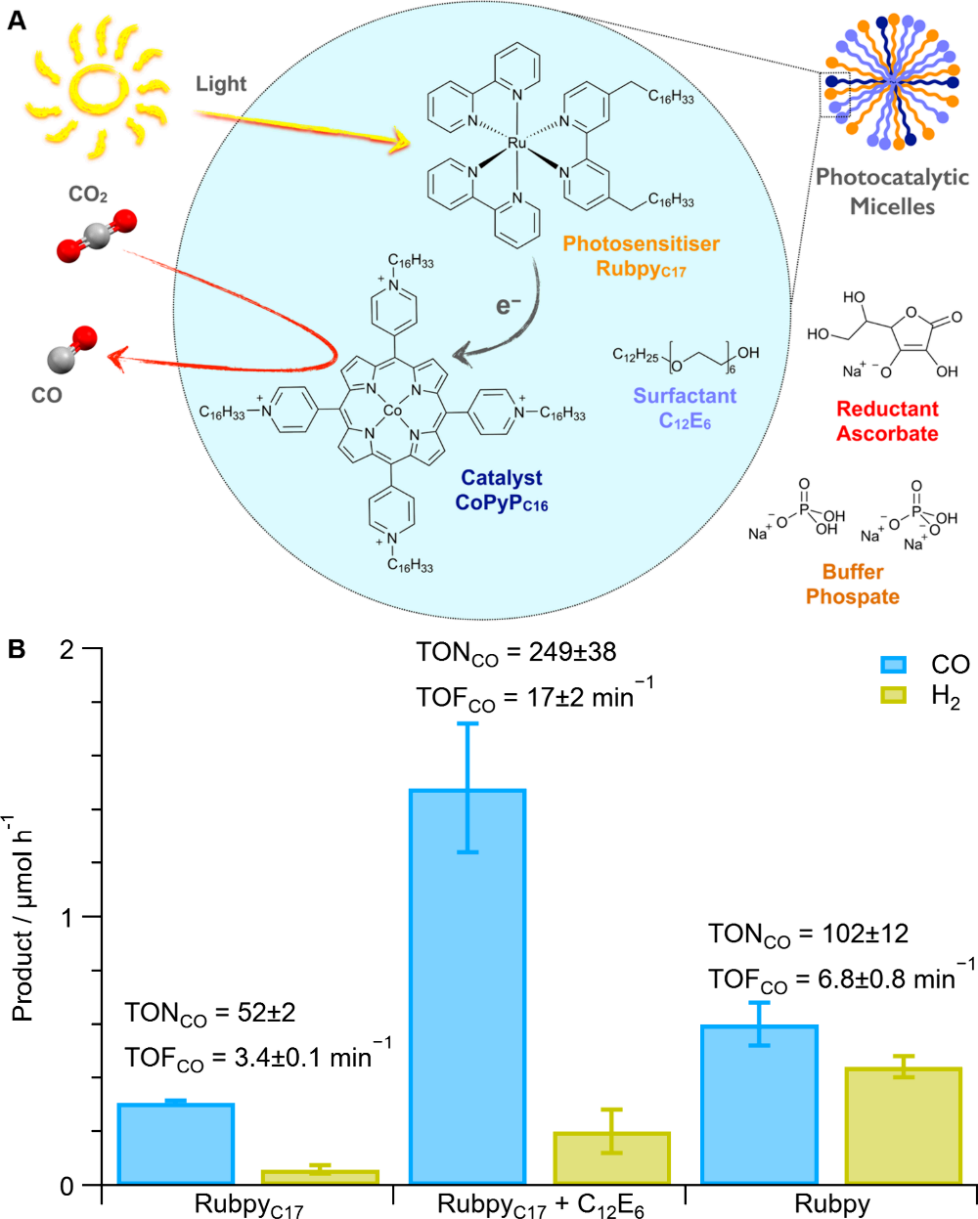
(A) Molecular components of photocatalytic system and reaction scheme; (B) photocatalytic test results with 1.5 μM CoPyP_C16_, 30 μM Rubpy_C17_ or [Ru(bpy)_3_]Cl_2_ (Rubpy), including or excluding 225 μM C_12_E_6_ (∼3 CMC), 100 mM NaHAsc, phosphate (0.1 M), CO_2_-sat. (pH 6.3) after 15 min 447 nm illumination with 2.3 W LED at 25 °C with 250 rpm orbital shaking, 1 mL reaction volume. Product detection by GC, 3 replicates except Rubpy_C17_ + C_12_E_6_ where *n* = 21 over 8 batches. Tabulated values in Table S1.

Systems that self-assemble into functional supramolecular structures are attractive targets, but they are also hard to optimize due to the numerous interdependent variables. This is exemplified herein with amphiphilic self-assembly of molecular components for photocatalytic CO_2_ reduction. The system exploits the tuneability of molecular components to functionalize a CO_2_ reduction catalyst and photosensitizer with alkyl tails and enable supramolecular assembly into micelles. The catalyst and photosensitizer are cationic complexes, to which alkyl tails were added to the periphery to render them amphiphilic while preserving the metal coordination environment and functionality.^16^ For the catalyst, cobalt tetra-methylpyridinium porphyrin (CoPyP_C1_) is an active catalyst for CO_2_-to-CO conversion in aqueous media,^21,22^ and was made amphiphilic by replacing the methyl groups with hexadecyl groups (CoPyP_C16_; Figure 1).^14^ A ruthenium tris-bipyridine was selected as it is a prototypical photosensitizer previously used for photocatalytic CO_2_ reduction with cobalt porphyrins and amphiphilic variants are known.^14,16,23^ To minimize additional functional groups, one bipyridine ligand was functionalized with two heptadecyl groups (Rubpy_C17_; Figure 1).^23^ In both cases, characterization of the complexes was consistent with previous reports and the UV–vis spectra showed minimal changes to λ_max_ to indicate that the coordination environment was preserved (details in Experimental Section). For photocatalytic studies, the water-insoluble [CoPyP_C16_](PF_6_)_4_ was solubilized in micelles from a MeCN stock solution into aqueous CO_2_-saturated phosphate solution (pH 6.3; MeCN removed in CO_2_ purging step). The solution also contained the Rubpy_C17_ photosensitizer and sodium ascorbate as a sacrificial electron donor (reductive quencher of the photosensitizer^14,21,24^); test conditions were 15 min illumination of 1 mL solution at λ = 447 nm using a 2.3 W LED at 25 °C with orbital convection.

The photosensitizer commonly serves as a single electron donor while two electrons are required for CO_2_-to-CO conversion, which has led to the established practice to have excess photosensitizer in photocatalytic tests, with 20–50 times excess previously used.^14,21,24^ Initial photocatalytic tests herein used 20:1 Rubpy_C17_ to CoPyP_C16_, which resulted in 52 ± 2 catalyst turnovers for CO formation (TON_CO_) to give a turnover frequency (TOF_CO_) of 3.4 ± 0.1 min^–1^ with selectivity for CO over H_2_ (Sel_CO_) of 84 ± 3% (Figure 1, Table S1; see SI Note 1 for list of metrics and definitions). This is more selective than literature values for the water-soluble catalyst variant (CoPyP_C1_) in aqueous phosphate buffer (Sel_CO_ 11%, 89% H_2_ formation),^14^ and comparable to a self-assembled liposome system (Sel_CO_ 77%).^14^ Ru photosensitizers are known to self-quench at high concentrations, which was mitigated herein by including the charge-neutral photocatalytically inactive surfactant hexaethylene glycol monododecyl ether (C_12_E_6_; critical micelle concentration, CMC, in H_2_O = 75 μM;^25^Figure 1). The inclusion of the nominally inactive C_12_E_6_ surfactant increased the TON_CO_ 5-fold to 249 ± 38 for a TOF_CO_ of 17 ± 2 min^–1^ and Sel_CO_ of 87 ± 2% (Figure 1), thereby demonstrating the importance of dispersing the photosensitizers in micelles to minimize self-quenching.

Comparing this result to other self-assembled systems that use alkylated Ru polypyridyl photosensitizers, the TOF_CO_ exceeds that of Lehn-type Re based systems ([Re^I^(bpy)(CO)_3_Cl]) in liposomes (17 ± 2 vs 0.08 or 1.1 min^–1^),^26,27^ and the CoPyP_C16_ catalyst in liposomes (0.8 min^–1^ at 0.5 μM or 6.1 min^–1^ at 0.02 μM).^14^ The micelle system also shows a comparable TOF_CO_ to a state-of-the-art quantum dot sensitized system that electrostatically assembles with a Co porphyrin in aqueous medium (13 min^–1^).^28^

Isotopic labeling with ^13^CO_2_ reveals that only ^13^CO is formed, confirming that all CO is formed from CO_2_ (Figure S1). Exclusion controls show that the system is inactive if any of CO_2_, catalyst, photosensitizer, reductant or light are removed (Figure S2). Amphiphilic Rubpy_C17_ results in a 2.4-fold higher TOF_CO_ and 30% higher CO selectivity than using its water-soluble hydrophilic analogue [Ru(bpy)_3_]Cl_2_ (Rubpy hereinafter, TOF_CO_ of 6.8 ± 0.8 min^–1^; Figure 1), demonstrating the activity enhancement enabled by supramolecular self-assembly of the micellar system. This self-assembly benefit is also observed when comparing to homogeneous photocatalytic systems in the literature using Co porphyrins in water, which reach comparable TOF_CO_ values (17 and 20 min^–1^) but use a 17-fold higher Ru photosensitizer concentration (30 vs 500 μM).^21,24^ This is likely due to higher local concentrations of photosensitizer in the micelle, thereby facilitating better use of the system components.

In contrast to nonionic C_12_E_6_, the use of cationic cetrimonium bromide (CTAB) and anionic sodium dodecyl sulfate (SDS) surfactants inhibited catalytic turnover, with no CO detected after equivalent measurements screening from 1 to 18-fold their nominal CMCs of 1.0 and 7.8 mM for CTAB and SDS in pure water,^29^ respectively (Figure S3 incl. chemical structures). UV–vis spectra measured before and after photocatalytic tests with and without C_12_E_6_ show the Rubpy_C17_-band intensity (λ_max_ 456 nm) decreases over time and correlates with decreasing catalytic turnover (Figure S4), indicating that the intrinsic instability of Rubpy complexes through photobleaching limits long-term performance and highlights the need to move beyond Ru-polypyridyl based photosensitizers.^21,22,28^ In contrast to using C_12_E_6_, no degradation of Rubpy_C17_ is observed when using CTAB or SDS surfactants within the time scale of the experiments (Figure S3), indicating that the ionic surfactants are inhibiting catalysis by preventing formation of long-lived reduced Ru states that are the most vulnerable to degradation.^21,30,31^

### Photoluminescence (PL) and Transient Absorption Spectroscopy (TAS)

**Figure 2:**
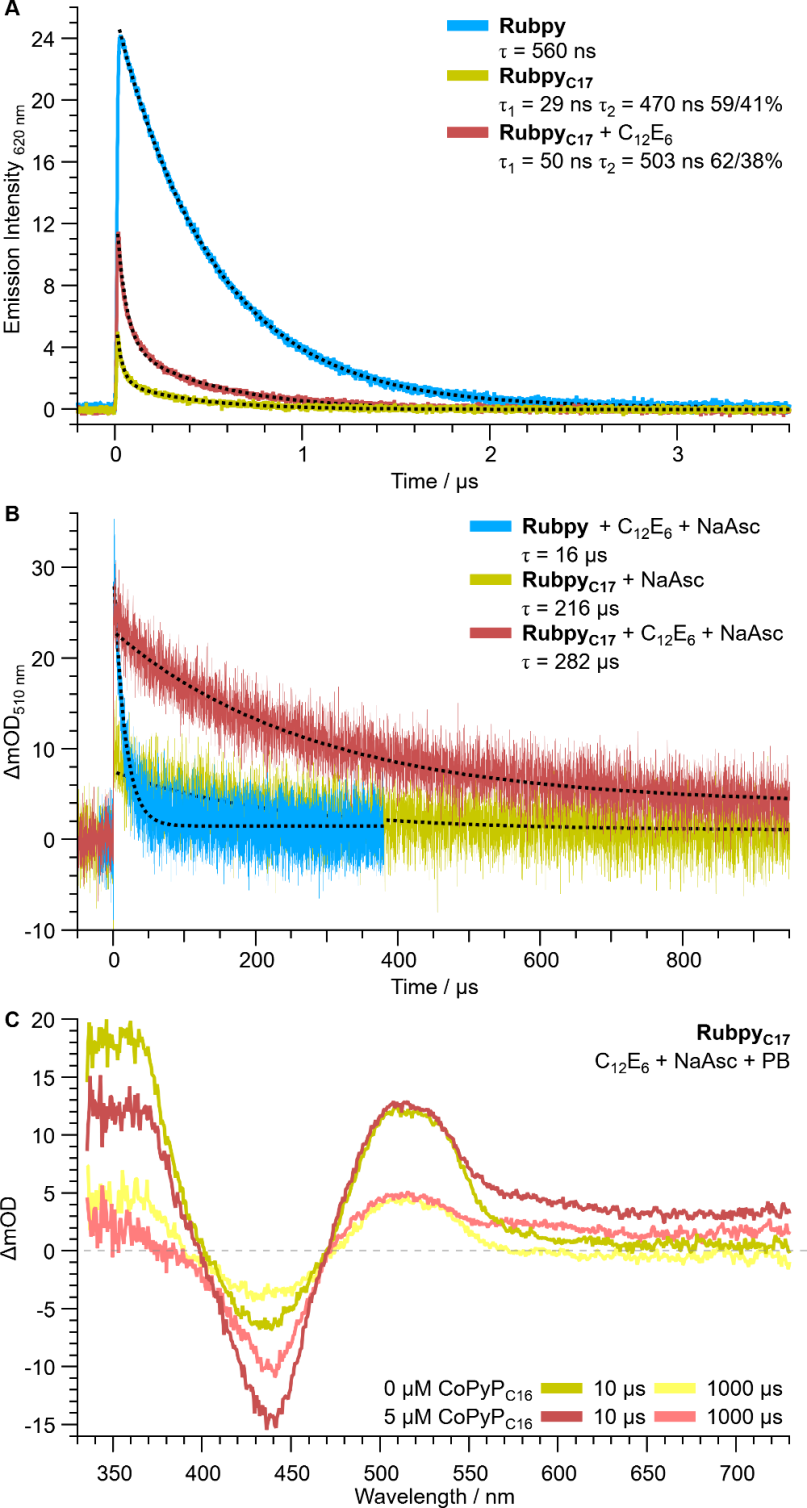
(A) Photoluminescence decay showing lifetime of excited photosensitizer in absence of reductant (excitation 460 nm, detection 620 nm); (B) transient absorption spectroscopy (pump 460 nm, probe 510 nm) measuring lifetime of reductively quenched photosensitizer in the presence of reductant; and (C) transient absorption spectroscopy (pump 460 nm) measuring spectral changes after 10 and 1000 μs delay. Thirty μM photosensitizer in phosphate buffer (PB, 0.1 M, pH 7.0), Ar purged. Five μM CoPyP_C16_, 225 μM C_12_E_6_ and/or 100 mM NaHAsc as indicated. Dashed lines show data fitting to determine lifetimes.

PL and TAS were employed to understand the charge-transfer processes of the self-assembled system and lifetime of transient photosensitizer states. Visible excitation of Rubpy complexes results in a metal-to-ligand charge transfer (MLCT) to give the charge-separated excited state [Ru^III^(bpy^•–^)(bpy)_2_]^2+^ (Ru*).^14^ Tracking the Ru* PL at 620 nm shows that C_12_E_6_ increases the Rubpy_C17_* lifetime, but does not affect the hydrophilic Rubpy* (Figure 2A, S5–6 and Table S2–3). This longer Rubpy_C17_* lifetime is attributed to C_12_E_6_ decreasing the local Rubpy_C17_ concentration in micelles and thereby limiting self-quenching and allowing more time for reductive quenching by ascorbate, which reduces the Ru^III^ center of Ru* to form [Ru^II^(bpy^•–^)(bpy)_2_]^+^ (denoted Ru^–^).^14^

Tracking Ru^–^ formation with TAS (510 nm probe) shows that adding C_12_E_6_ increases the Rubpy_C17_^–^ yield by 50%, resulting in a quenched photosensitizer yield that is only 25% lower than diffusionally free Rubpy^–^ (17 to 30 vs 40 ΔmOD for Rubpy_C17_, Rubpy_C17_+C_12_E_6_ vs Rubpy; Figure S7 and Table S4). The Rubpy_C17_^–^ lifetime is then 18-fold longer than Rubpy^–^ with C_12_E_6_ and phosphate buffer (282 vs 16 μs; Figure 2B, Figures S8 and S9 and Table S5), allowing more time for electron transfer to the catalyst.

Adding CoPyP_C16_ results in deeper absorbance bleaching because its Soret band (λ_max_ 440 nm; Figure S10) is convoluted with Rubpy (λ_max_ 456 nm). However, CoPyP_C16_ increases the Rubpy_C17_^–^ lifetime by 33% rather than oxidizing it (282 to 375 μs at 510 nm and lack of bpy^•–^ decay at 366 nm; Figure 2C and Figure S11). The rapid formation of CoPyP_C16_^–^ (broad response at 550–750 nm within 10 μs),^14^ the longer Rubpy_C17_^–^ lifetime, and the relevant redox potentials indicates that HAsc^–^ is oxidized by Rubpy_C17_* to form Asc^•^, which then reduces CoPyP_C16_ and forms dehydroascorbate.^32^ This pathway prevents charge recombination between Asc^•^ and Rubpy_C17_^–^ by forming CoPyP_C16_^–^. It also suggests that the system extracts two electrons from the donor per photon, with the process enabled by the high local concentrations inside the micelles. Further TAS analysis including for the ionic surfactants is provided in the Supporting Information (SI Note 2).

### Heuristic human optimization

**Figure 3:**
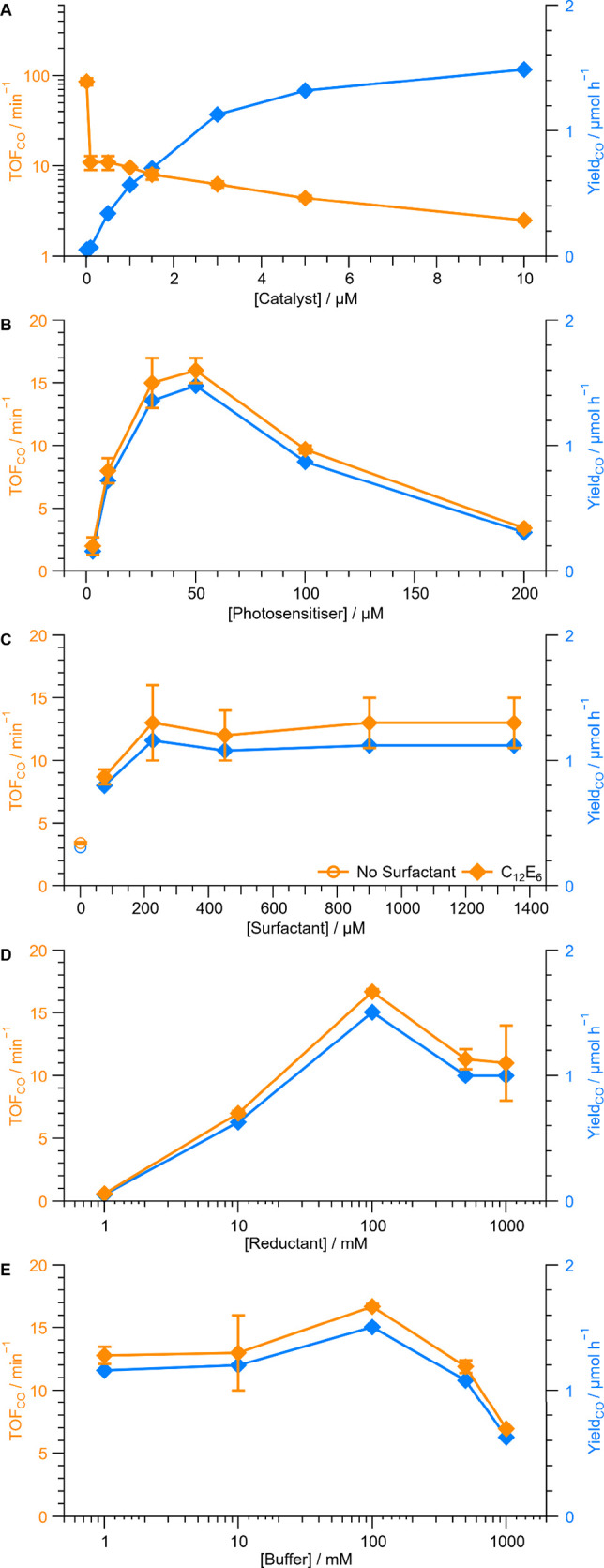
Heuristic optimization of photocatalytic system showing catalyst turnover frequency (TOF) and Yield of CO as a function of catalyst (A), photosensitizer (B), surfactant (C), reductant (D) and buffer (E) concentration. Unless specified, 1.5 μM CoPyP_C16_, 30 μM Rubpy_C17_, 225 μM C_12_E_6_ (∼3 CMC), 100 mM NaHAsc, phosphate (0.1 M), CO_2_-sat. (pH 6.3) after 15 min (60 min for catalyst series) 447 nm illumination with 2.3 W LED at 25 °C with 250 rpm orbital shaking, 1 mL volume, GC quantification, 3 replicates.

The photocatalytic system has categorical variables (variables that are divided into groups, often non-numerical such as the molecular structures) and continuous variables (a quantitative variable that can be any value with a fixed range, e.g., concentrations). Five continuous variables were sequentially varied in the system, namely, concentrations of catalyst, photosensitizer, surfactant, reductant and buffer, while the performance was quantified with five figures of merit, namely, Yield_CO_ (moles of CO formed), QY_CO_ (percentage of incident photons forming CO), TON_CO_ (the number of catalyst turnovers forming CO), TOF_CO_ (TON_CO_ over time), and Sel_CO_ (the percentage of CO formed from the sum of CO and H_2_ formed) to determine the sensitivity of each metric to the variables and start building the algorithm training set (Figure 3 and Table S6). Because the experiment time is fixed, Yield_CO_ and QY_CO_ must follow the same trends, as must TON_CO_ and TOF_CO_. Varying the catalyst concentration shows that lower catalyst concentrations allow observation of higher TOF_CO_ (∼86 ± 8, 11 ± 2, 2.5 ± 0.1 min^–1^ at 0.01, 0.1, and 10 μM, respectively; Figure 3A); a trend that is consistent with general observations in the literature.^14,21,22,24^ However, the high TON and TOF come at a cost of selectivity and product yield (25, 50 and 89% CO and 0.05, 0.07, and 1.5 μmol CO h^–1^), emphasizing the importance of considering the system holistically rather than focusing on individual figures of merit.

Variation of the photosensitizer concentration shows a peak TOF_CO_ at 17 ± 2 min^–1^ for 30 μM with clear decays at higher or lower concentration (2.0 ± 0.7 at 3 μM and 3.4 ± 0.2 at 200 μM; Figure 3B) with constant CO selectivity (88 ± 1%). The peak performance occurs with concentrations able to effectively absorb light but is far below full absorption (*A*_456_ = 0.39), which may be due to a lack of surfactant to prevent self-quenching, lack of catalyst or reductant or a combination thereof. Keeping the photosensitizer concentration constant and increasing the C_12_E_6_ surfactant concentration from 0 to 225 μM (3 CMC) increases the TOF_CO_ followed by a plateau with constant 89 ± 1% selectivity (Figure 3C). TOF_CO_ increases with the concentration of the ascorbate reductant (0.6 ± 0.1, 17 ± 2, 11 ± 3 min^–1^ at 1, 100, 1000 mM, respectively; Figure 3D), with the lowest concentrations limiting photosensitizer quenching whereas the highest likely increase back-reactions.^33^ With these fixed concentrations of the other components, the phosphate concentration had a minimal effect until performance decreased as surfactant solubility was visibly lowered (TOF_CO_ 12.8 ± 0.7, 17 ± 2, 7 min^–1^ at 1, 100, 1000 mM respectively. Figure 3E). In contrast to the catalyst concentration, the trend of the Yield_CO_ follows the TOF_CO_ for the photosensitizer, surfactant, reductant and buffer concentrations. However, it is not clear how to best increase both the Yield_CO_ and TOF_CO_ for the system. Notably, the reductant and buffer concentrations significantly affect the solution ionic strength to indirectly influence micelle size and shape, as does the loading of catalyst and photosensitizer, yet varying the concentrations individually cannot capture these effects. Fully exploring the parameter space with 10 concentrations of each of the 5 variables would require a humanly impractical 10^5^ experiments (SI Note 3). Thus, we employed Bayesian statistics to bring the number of experiments to an actionable number for a human experimentalist.

### Learning algorithm (Bayesian) optimization

**Figure 4:**
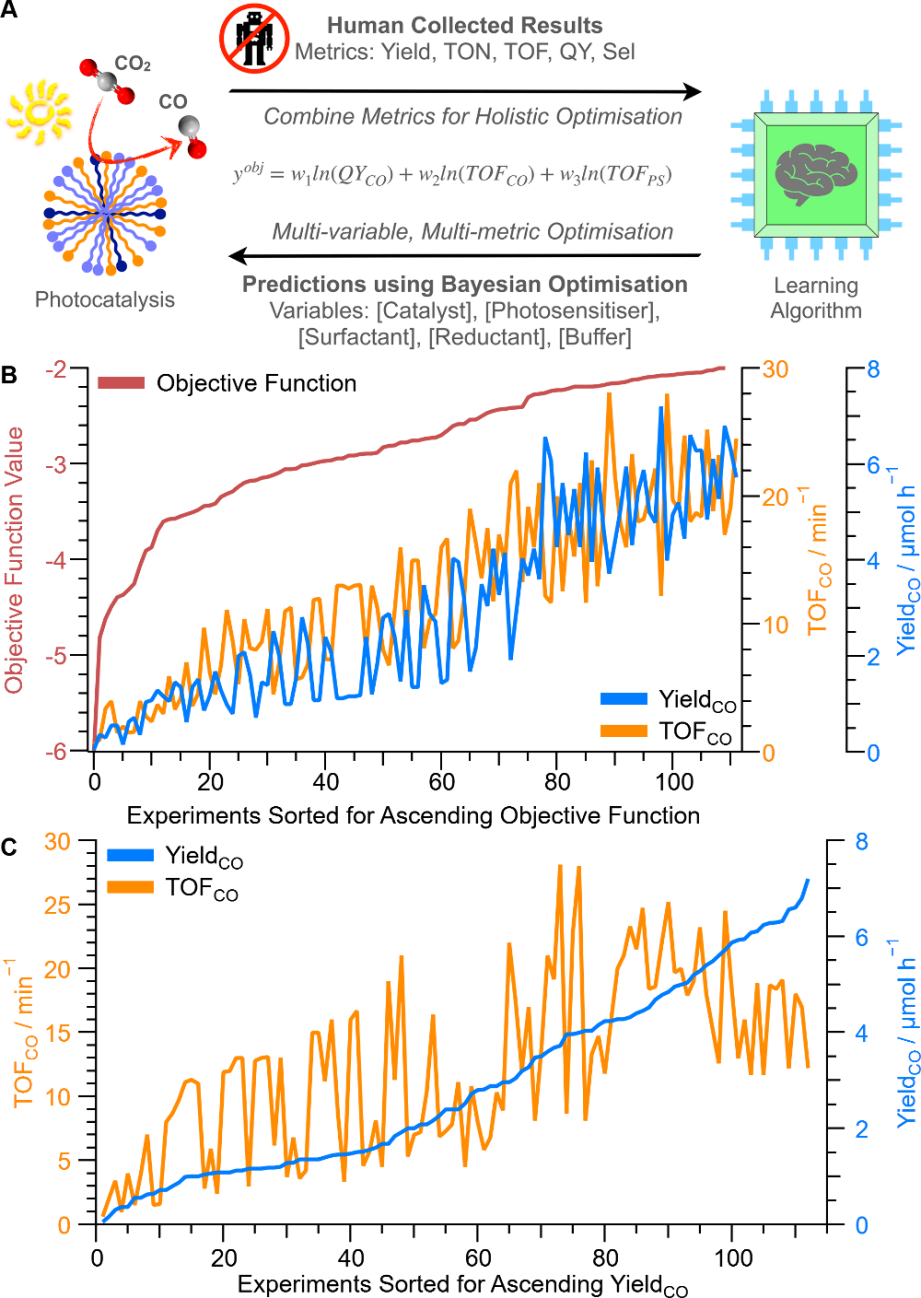
Holistic optimization using learning algorithms. Overview of the workflow (A); sorted data to show holistic improvement measured by objective function (eq 1 with *w*_1_ = 0.4, *w*_2_ = 0.4, *w*_3_ = 0.2) alongside improvement of both Yield_CO_ and TOF_CO_ (B); and sorted data showing ascending Yield_CO_ and corresponding TOF_CO_ (C). The data in B and C are sorted and not in chronological order. Full experimental results tabulated in Table S7. Experimental conditions: various concentrations of CoPyP_C16_, Rubpy_C17_, C_12_E_6_, NaHAsc and phosphate buffer, CO_2_-sat. (pH 6.3) under 447 nm illumination with 2.3 W LED at 25 °C with 250 rpm orbital shaking, 1 mL reaction volume for 15 min, GC quantification.

Our approach enables variation of *>*3 parameters simultaneously to increase optimization speed and find relationships between interdependent parameters, thereby allowing rapid and simultaneous optimization of multiple performance metrics. Our objective function (eq 1 and Figure 4) provides a single metric that incorporates key figures of merit to quantify overall system performance (holistic performance) and is defined as the sum of the weighted natural logarithms of QY_CO_, TOF_CO_, and TOF_PS_ to balance a high product yield against high efficiency. Sel_CO_ is indirectly considered by the QY_CO_ and TOF_CO_. *w*_1_, *w*_2_ and *w*_3_ are weighting coefficients to define the relative importance of each metric.
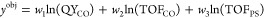
1

The workflow comprised iterative experimental testing, from which the algorithm learnt and predicted new conditions (Figure 4A or Figure S12 for a more technical description). The aim was to maximize the objective function (eq 1), which represents overall performance. The process is (i) the algorithm learns from the initial data set pool (subset of heuristic optimization data, Table S7); (ii) the algorithm predicts the next values to test; (iii) the data set pool is enlarged with new results; (iv) new predictions and iteration.^34^ Two Bayesian optimization methodologies (GpyOpt^35^ and Gryffin^36^) were used with multiple acquisition functions to control paths through promising regions of the parameter space and exploit known peaks, while balancing the need to explore new regions (exploitation vs exploration; see Experimental Section). GpyOpt and Gryffin were selected as they have shown excellent performance in reaching the global optimum among Bayesian Optimization methods,^36^ which are already superior to optimizations based on local gradient and Hessian approximations.^34^ Holistic optimization proceeded through 10 iterations with each iteration providing five concentrations of catalyst, photosensitizer, surfactant, reductant, and buffer to test (Figure 4; each prediction is the mean of 5 replicate algorithm runs which are shown with their standard deviations in Figure S13; the mean prediction values and experimental results are shown in Table S7). Two optimizations were conducted in parallel by using different weightings of the objective function components, where *y*^obj1^ considered all components with weighting coefficients *w*_1_ = 0.4, *w*_2_ = 0.4, *w*_3_ = 0.2; while *y*^obj2^ excluded the TOF_CO_ of photosensitizer (TOF_PS_) with *w*_1_ = 0.5, *w*_2_ = 0.5, *w*_3_ = 0. Equal weighting was given to the QY_CO_ and TOF_CO_ to prioritize maximization of both metrics simultaneously (the selection of weights is subjective as it depends on the prioritization of metrics meaning there is no mathematically correct choice). By optimizing the two objective functions simultaneously, this provides 10 sets of conditions (optimization paths) each iteration, which matches the capacity of the experimental equipment. The results were then all added into the data set pool to provide all possible information to all algorithms (largest possible data set pool at each iteration).

The optimizations including and ignoring the photosensitizer (*y*^obj1^ and *y*^obj2^) both reached maxima with the same conditions in iteration 8/10, and displayed improvement in all figures of merit included in the objective function. Compared to the intuitively selected and literature supported starting conditions that appeared high performing based on initial tests and heuristic optimization (Table 1), the holistically optimized conditions displayed 4.2-fold higher Yield_CO_ (5.7 ± 0.1 vs 1.5 ± 0.2 μmol h^–1^), 57% higher TON_CO_ (368 ± 8 vs 249 ± 38), 47% higher TOF_CO_ (25 ± 1 vs 17 ± 2 min^–1^) and 3.8-fold higher QY_CO_ (0.15 ± 0.01 vs 0.04 ± 0.01%) while Sel_CO_ was unchanged (88 vs 87 ± 2%). This shows that systems can be optimized for high Yield, TON, TOF and QY rather than focusing on a single metric. These values are exceptional for photocatalytic CO_2_-to-CO reduction in aqueous media with a far higher TOF_CO_ than Lehn-type Re complexes in liposomes,^26,27^ higher TOF_CO_ and Sel_CO_ than high performing homogeneous Co porphyrin systems that use 17-fold higher photosensitizer concentration,^21,22,24^ and higher TOF_CO_ than even a high performing quantum dot sensitized Co porphyrin system (Table S8).^28^ Expectedly, the objective function (overall system performance metric) is not linearly correlated with any of the system components (r^2^ < 0.27 in all cases; full linear correlation plots and correlation coefficient matrix in Figure S14 and S15). The highest correlation coefficient of a parameter with the objective function is 0.27 for [photosensitizer] and 0.17 for [surfactant], while correlations of the objective function with other parameters have coefficients <0.05. This confirms that increasing all parameters would not result in the observed performance improvement. TAS reveals that these conditions coincide with the Rubpy_C17_^–^ lifetime shortening from 375 to 190 μs in the optimized system, suggesting more efficient electron transfer to the catalyst (Figure S16).

**Table 1:** Original and optimized figures of merit for photocatalytic system composed of CoPyP_C16_, Rubpy_C17_, C_12_E_6_, NaHAsc, phosphate buffer, CO_2_-sat. (pH 6.3) under 447 nm illumination with 2.3 W LED at 25 °C with 250 rpm orbital shaking, 1 mL reaction volume for 15 min, GC quantification^a^

	**original**	**holistic opt.**	**max Yield**_**CO**_**& QY**_**CO**_^b^	**max TON**_**CO**_**& TOF**_**CO**_^b^
**Yield**_**CO**_**/μmol h**^**–1**^	1.5 ± 0.2	5.7 ± 0.1	7.20 ± 0.04	3.72 ± 0.08
**TON**_**CO**_	249 ± 38	368 ± 8	184 ± 8	422 ± 8
**TOF**_**CO**_**/min**^**–1**^	17 ± 2	25 ± 1	12 ± 1	28 ± 1
**QY**_**CO**_	0.04 ± 0.01	0.15 ± 0.01	0.19 ± 0.01	0.10 ± 0.01
**[Cat]/μM**	1.5	3.9	9.8	2.2
**[PS]/μM**	30	112	98	109
**[Surf.]/CMC**	3	19	25	32
**[Red.]/mM**	100	220	348	195
**[Buf.]/mM**	100	462	323	509

a 21 replicates over 8 batches for “original”, 3 replicates for others.

b Maximum values show performance and conditions that resulted in the highest Yield_CO_ and QY_CO_ or highest TON_CO_ and TOF_CO_ during the optimization. Yield_CO_ and QY_CO_ are linked, as are TON_CO_ and TOF_CO_, because the reaction time is constant meaning they must be maximized under the same conditions.

Sorting the experiments shows that all the performance metrics improved as the objective function was maximized (Figure 4B), thereby validating the way the objective function was defined and showing a stark contrast to the response from heuristically varying the catalyst concentration (Figure 3A). However, neither the highest Yield_CO_ and QY_CO_ nor highest TON_CO_ and TOF_CO_ were obtained at the holistic optimum because a compromise in conditions is required (Table 1). To reiterate, Yield_CO_ and QY_CO_ are linearly correlated (Figure S14 and S15), as are TON_CO_ and TOF_CO_, because QY is Yield over a constant and TOF is TON over a constant (with reaction time and photon flux being constant), hence they are maximized under the same conditions. At conditions for the highest Yield_CO_ and QY_CO_ (7.20 ± 0.04 μmol h^–1^ and 0.19 ± 0.01%), the TON_CO_ and TOF_CO_ are ∼50% lower than the holistic maximum (184 ± 8 and 12 ± 1 min^–1^; Figure 4C). Coherently, conditions displaying the highest TON_CO_ and TOF_CO_ (422 ± 8 and 28 ± 1 min^–1^) result in Yield_CO_ and QY_CO_ being ∼50% lower (3.72 ± 0.08 μmol h^–1^ and 0.10 ± 0.01%) than their ideal conditions. In other words, TOF_CO_ and Yield_CO_ are not linearly correlated, especially at high values, meaning that these maxima cannot be reached by maximizing only one metric. This demonstrates how objective functions enable holistic optimization and highlights the limitations in focusing on individual figures of merit as remains prevalent in the literature.

### Post-Optimization Machine Learning Analysis

**Figure 5:**
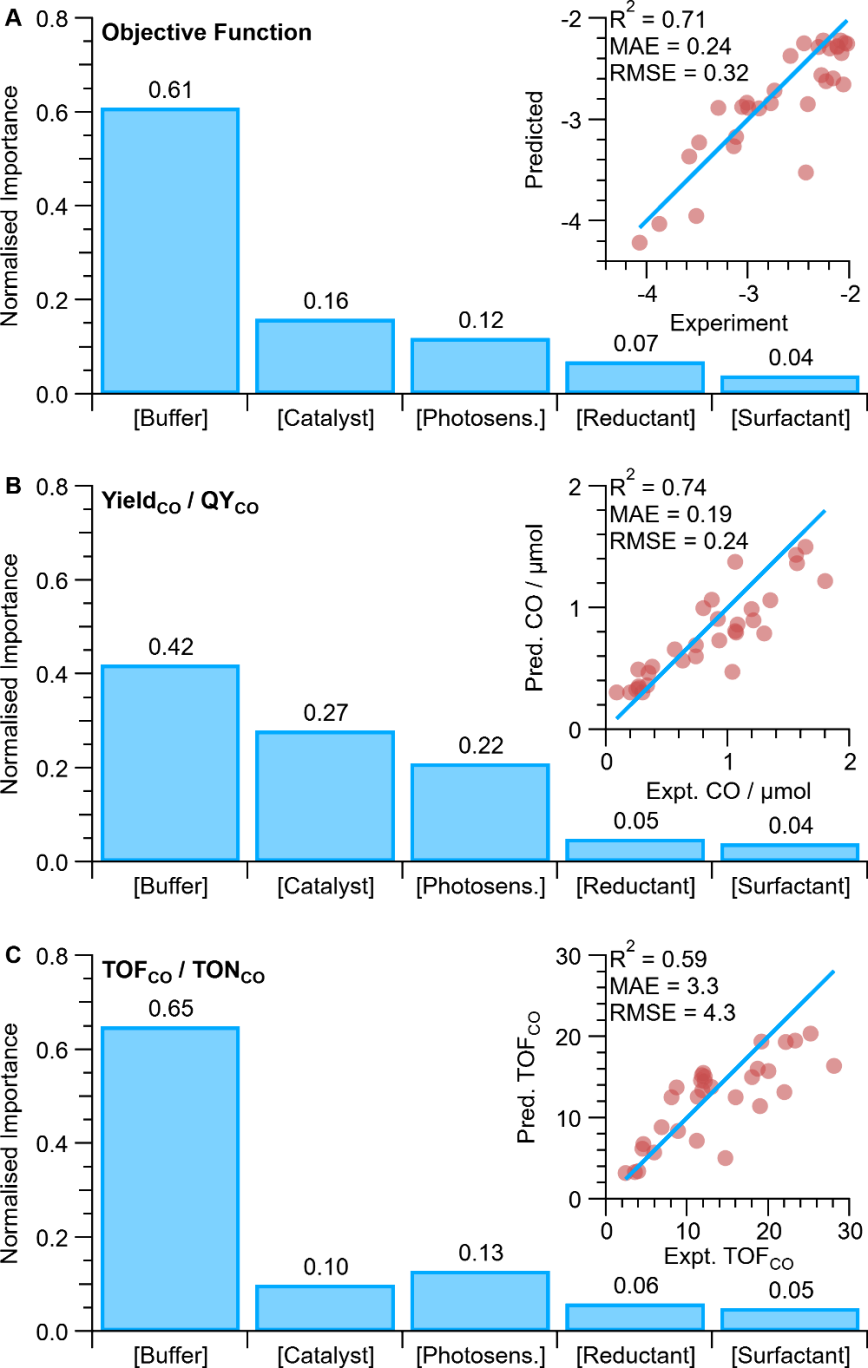
Machine learning analysis showing the normalized importance of each system component to holistic optimization by objective function 1 (A), Yield_CO_ and QY_CO_ (B), TON_CO_ and TOF_CO_ (C); with machine learning predictions with the Random-forest regression models (inset), where model performance is shown by coefficient of determination *R*^2^, mean absolute error (MAE), and Root Mean Squared Error (RMSE).

The internally consistent data set produced during the optimization was then exploited to determine the ruling parameters for each metric with machine learning algorithms, i.e., the quantitative importance of each parameter to overall system performance and to individual figures of merit was extracted. The full data set was split into two sections, with 70% used to train/cross-validate the regression model while the model predicted results for the remaining 30%, thereby allowing the model to be tested against experimental results (Random Forest with *k*-fold cross-validation was employed to identify the most effective hyperparameters for the regression models; see SI Note 4).^37,38^ The number of data points is low by machine learning standards with only 72 experiments for training/cross-validation and 31 experiments for testing, yet the consistency of the data set means the regression model is accurately predictive of how the concentration of each component will affect holistic system performance (*R*^2^ = 0.71 is suitable for a predictive model trained on experimental data;^10,39^Figure 5A).

Shapley additive explanations (SHAP) were then employed to quantify the importance of each parameter (system component) on the system performance (Figure 5A),^17−19^ revealing the buffer concentration as the parameter with dominating importance (61%), far more than the catalyst or photosensitizer concentrations (16 and 12%, respectively; note the 4% difference may not be statistically significant). The nonlinear effect of the buffer concentration is also visible in the correlation pair-plot while other parameters show more stochastic scatter plots (Figure S14). While the buffer concentration allows the ionic strength to be varied and thereby provides control of micelle size, its importance is seemingly contradictory to the heuristic optimization wherein the concentration of buffer did not affect the system until it lowered solubility and hindered performance (Figure 3E). However, the ubiquitous heuristic optimization did not consider dependencies between the variables, thereby exemplifying the value of the statistics driven holistic optimization demonstrated herein. Consequently, SHAP analysis can quantitatively identify the cause of improvements after Bayesian Optimization, which provides insight that enables rationalization and further system development. It is especially valuable as the causes identified may not be immediately visible to the experimentalist.

Machine learning regression and the SHAP routine then extracted the importance of each parameter to individual figures of merit (Figure 5B,C), thereby providing insight into the conditions that favor high Yield_CO_ and QY_CO_ vs those that favor high TON_CO_ and TOF_CO_. In all cases the buffer concentration remains the most important factor, with comparably dominating importance to TON_CO_ and TOF_CO_ (64–65%), yet buffer concentration is far less important to Yield_CO_ and QY_CO_ (42–43%) where the catalyst and photosensitizer concentrations grow in importance (27 and 22%, respectively). Thereby, the conflicting requirements to have high Yield_CO_ vs high TOF_CO_ can be quantitatively linked to different parameter combinations. Consistent with these results, maximum CO yield is reached with 4.5-fold higher catalyst concentration than maximum TOF_CO_ (9.8 vs 2.2 μM), whereas higher buffer concentration is required for maximum TOF_CO_ (509 vs 323 mM). The holistically optimized results balance these two requirements, yet a similar photosensitizer concentration is maintained for all three maxima, presumably to maximize light absorption and quantum yield.

### Control Group Feature Analysis

Further leveraging the holistic optimization data set, local performance maxima in the 5-dimensional parameter space can be identified to find conditions that significantly reduce the amount of valuable components used at a minor cost to system performance. These fruitful regions of the parameter space can be identified and visualized by reducing the five variables into a lower number of features following the recently introduced Control Group Feature analysis (herein, the two mixed features are defined as x_1_ and x_2_ in Figure S17; details in Experimental Section).^20^ The approach is inspired by Buckingham analysis and based on the principle that variable combinations that offer the same mixed feature value will offer comparable performance by exploiting alternate maxima in the parameter space. This analysis highlights regions of the parameter matrix containing high performance conditions as clustered high-performing values in scatter plots.

The Control Group Feature analysis shows clustered regions for values of the holistically optimized system (objective function), Yield_CO_ and QY_CO_, but no clear regions were found for TON_CO_ and TOF_CO_ (Figure S17). Focusing on the holistic performance, alternative parameter combinations (component concentrations) were identified that are predicted to provide comparable performance while using 30% less catalyst and 16% less photosensitizer (Catalyst 2.7 vs 3.9 μM; Photosensitizer 94 vs 112 μM; Surfactant 23 vs 19 CMC; Reductant 256 vs 220 mM; Buffer 402 vs 462 mM). Thereby, the data set is further exploited to minimize the more expensive, metal-containing components at a minor performance cost, which is a key trade-off for system commercialization (additional analysis in SI Note 5).

### Wider Implications

Methodology for multivariable multimetric optimization is required in many fields to move beyond the focus on individual figures of merit that drives optimization away from practical conditions. Overall system development requires multiple metrics to be improved simultaneously, even though some metrics initially appear to require opposite conditions, e.g., high TOF observed at low catalyst loading while high product yield requires the opposite as initially observed in this study. Even pioneering high-throughput robotic approaches have focused on single figures of merit,^6,40^ including those employing machine learning.^5,13,41^ New approaches should minimize expensive and space-demanding equipment wherever possible to facilitate widespread adoption, which is a barrier that continues to hinder high-throughput robotic setups. Learning algorithms require no elaborate equipment and can accelerate multivariable optimization so effectively that human experimentalists can complete otherwise insurmountable studies, such as multimetric optimization of complex systems. The use of overall performance metrics can take into account a variety of factors, for example, using an expensive component will be justified if the performance improvement outweighs the cost and leads to an improvement in the overall metric. Thereby such comparisons can be objective when comparing systems and conditions. To fully exploit this approach, each field should reach a consensus on a standardized metric against which all systems should be compared. In some ways, this could be a powerful extension of the approach taken for solar cell characterization where photon to current efficiency is the standard metric of comparison.

The highly general methodology for holistic optimization shown herein can be applied in any chemistry laboratory to address the inability to complete multivariable multimetric optimization in many fields beyond photocatalytic CO_2_ reduction (incl. materials, polymer and organic synthesis). While the approach is demonstrated with CO_2_ reduction, a clear example application is optimizing organic synthesis reactions so they are high yielding, fast and general (wide substrate scope), i.e., multivariable multimetric reaction optimization is required.^6^ For an organic reaction, postoptimization machine learning would then quantify the importance of each variable to each metric and guide mechanistic understanding; e.g., the importance of the buffer herein is analogous to additives that unexpectedly enhance organic reactions, which has been the focus of a prominent high-throughput study.^6^

## Conclusions

We demonstrate how to optimize multivariable multimetric systems using five-component self-assembled photocatalytic micelles for CO_2_-to-CO reduction as a test platform. Self-assembly improved the system’s photocatalytic performance 2.4-fold compared to freely diffusing components, with transient absorption spectroscopy showing this was due to an 18-fold longer reduced photosensitizer lifetime. However, standard optimization showed that the system could not be optimized for both high TOF_CO_ and high CO yield as they appeared to require opposite conditions. Therefore, we established a novel approach and defined a holistic metric set that encompasses overall system performance and which was optimized using machine learning algorithms to simultaneously improve CO yield (4.2-fold), TON_CO_ (57%), TOF_CO_ (47%) and quantum yield (3.8-fold), while maintaining 87% CO selectivity over H_2_. Machine learning enabled this optimization in 103 tests compared to 10^5^ combinatorial possibilities, thereby bringing multivariable multimetric optimization into reach for all chemistry laboratories without expensive robotic or high-throughput setups.

A predictive machine learning model was then trained on the data set and quantified the effect of each component. This revealed surprisingly that the buffer has 61% importance to the holistic performance despite heuristic optimization indicating the buffer concentration had a marginal effect, thereby emphasizing the complexity of systems with interdependent variables. The buffer is 20% more important to TON_CO_/TOF_CO_ than it is to yield of CO and quantum yield, which demonstrates the trade-offs required for holistic optimization and why maximizing a single metric has limited meaning to overall system performance.

Future standardization of holistic performance metrics will enhance comparability and focus research on meaningful system performance rather than a subjective individual metric that often relies on impractical conditions. Exploiting developments in machine learning and data science will concurrently reveal system limitations and guide the understanding that underpins progress across research in catalysis.

## Experimental Section

### Reagents

Commercial chemicals were used as supplied: hexaethylene glycol monododecyl ether (C_12_E_6_; CAS 3055–96–7, Sigma, ≥ 98%); sodium phosphate, dibasic (CAS 7782–85–6, Thermo Scientific, ≥ 99%); sodium phosphate, monobasic (CAS 10049–21–5, ACROS, > 99%); sodium dodecyl sulfate (CAS 151–21–3, ThermoScientific, 99%); hexadecyltrimethylammonium bromide (CAS 57–09–0, ACROS, ≥ 99%); sodium L-ascorbate (CAS 134–03–2, Sigma, ≥ 99%); tris(2,2′-bipyridyl)ruthenium(II)dichloride hexahydrate (CAS 50525–27–4, Sigma, 99.95%); hexadecyltrimethylammonium chloride (CAS 112–02–7, Sigma, 25 wt.% aqueous solution). Deionized water was used throughout (18 MΩ cm, Milli-Q), MeCN was freshly distilled. All gases were obtained from BOC.

### Synthesis

**CoPyP**_**C16**_ was synthesized following reported procedures;^14^ briefly, tetrapyridyl-porphyrin (PyP), was reacted with excess C_16_H_33_Br at 130 °C for 16 h in anhydrous DMF under a N_2_ atmosphere to form the tetraalkylated porphyrin (PyP_C16_). Purification was by precipitation from CHCl_3_:MeOH (85:15) with acetone and reprecipitation from hot EtOH. EA: C, H, N values from all batches within 0.5% of calculated for C_104_H_158_N_8_Br_4_·3H_2_O (1894.1 g mol^–1^): C 65.95, H 8.73, N 5.92%. ^1^H NMR (DMSO-d_6_) and ^13^C NMR (DMSO-d_6_) as reported.^14^ Metalation of PyP_C16_ used excess Co(OAc)_2_ at 120 °C for 3 h in anhydrous DMF under a N_2_ atmosphere followed by precipitation with Et_2_O. CoPyP_C16_ was then precipitated from acetone:MeOH (9:1) with aqueous NaPF_6(sat’d)_, and reprecipitated from acetone with water. EA: C, H, N values from all batches within 0.5% of calculated for CoC_104_H_156_N_8_P_4_F_24_·3H_2_O (2211.3 g mol^–1^): C 56.59, H 7.38, N 5.07%. **Rubpy**_**C17**_ was synthesized following reported procedures from [Ru(Cl)_2_(bpy)_2_] and 4,4′-diheptadecyl-2,2′-bipyridine (bpy_C17_).^27,42^^1^H and ^13^C NMR (CH_3_OD) and UV–vis (CH_3_OH) as reported. EA: C, H, N values from all batches within 0.5% of calculated for RuC_64_H_92_N_6_Cl_2_·7H_2_O (1243.6 g mol^–1^): C 61.81, H 8.59, N 6.76%.

### Solution preparation

The water-soluble chloride salts of photosensitizers [Ru(bpy)_2_(bpy_C17_)](Cl)_2_ or [Ru(bpy)_3_](Cl)_2_ were prepared as aqueous stock solutions and used within 2 days. The PF_6_^–^ salt of CoPyP_C16_, [CoPyP_C16_](PF_6_)_4_, was prepared as an acetonitrile solution that was confirmed by UV–vis to be stable for months. Concentrations of stock solutions were determined by UV–vis (Agilent Cary 60) and known or determined molar extinction coefficients rather than relying on weighing small masses. Surfactant and ascorbate solutions were prepared fresh daily with concentrations based on weighed mass (excl. CTAC), while phosphate buffer was prepared as a larger stock from the monobasic and dibasic sodium salts (H_2_PO_4_^–^:HPO_4_^2–^ as 0.615:0.385 molar ratio to reach pH 7.0 in the final solution).^43^ Reaction solutions were prepared by diluting these stock solutions into water to reach the desired concentrations, with the minimal volume of organic solvent (MeCN) excluded from the final volume as it is removed during CO_2_ purging.

### Photocatalysis

One mL aliquots of solution, (3 mL for ^13^CO_2_ labeling experiment), were delivered into crimp-cap vials sealed with septa and purged through inlet and outlet needles for 15 min with CO_2_ or N_2_ including 2 vol.% CH_4_ as an internal standard. CO_2_ saturation was reached within 10 min and defined by the solution stabilizing at pH 6.3. The profile of the foam changes during purging as the MeCN is removed. The vials were then placed in a LED photoreactor (Treellum Technologies, Patent EP17382313) with a thermostatically controlled heating block at 25 °C with 250 rpm orbital convection; wherein the vials were illuminated with 447 nm LED light (20 nm spectral width) from 2.3 W LEDs (Luxeon Rebel ES LED “Royal Blue”).^44,45^ The reaction headspace was sampled via gastight syringe (100 μL aliquot; Hamilton) and analyzed by gas chromatography (Shimadzu Tracera GC-2010 Plus with a discharge ionization detector) using a molecular sieve column (Restek 5A PLOT, 0.53 mm ID, 30 m) at 85 °C. H_2_ and CO peak areas were converted to moles using calibrated response factors and the 2 vol.% CH_4_ internal standard. Quantum yields were calculated based on photon flux previously determined by actinometry for the photoreactor as 2.1 μmol photons s^–1^.^9^ Isotopic labeling used ^13^CO_2_ for photocatalysis followed by transferring the vial headspace into an evacuated gas infrared cell (SpecAc, 10 cm path length, KBr windows) and collecting a FT-IR transmission spectrum (ThermoScientific Nicolet iS50). Data processing used MatLab (Mathworks) while plotting used Igor Pro (Wavemetrics).

### Transient Absorption Spectroscopy

Nanosecond optical excitation (pump) used a Nd:YAG laser/OPO (EKSPLA NT 342) to deliver 8 mJ pulse^–1^ at 460 nm. The measurement (probe) spectrometer (Edinburgh Instruments LP 920-K) was equipped with a 450 W Xe arc lamp (Osram) and CCD camera (Oxford Instruments Andor iStar) connected to an oscilloscope (Tektronix TDS3052B). Measurements were through a 3 mL 10 × 10 mm quartz cuvette at ambient temperature after purging with Ar or CO_2_ where specified, with 20 scans averaged for each spectrum. Data fitting used Igor Pro (Wavemetrics).

### Bayesian Optimization

GpyOpt 1.2.6^35^ and Gryffin^36^ (dynamic implementation) methodologies were employed. Three acquisition functions were employed with GpyOpt: exploitation/exploration trade-off functions Expected Improvement (EI) and Lower Confidence Bound (LCB), and exploitation focused Maximum Probability of Improvement (MPI). Two acquisition functions were employed with Gryffin: exploitation and exploration (hyperparameter λ to +1 or −1, respectively). Predictions were made by taking the mean of 5 repeated queries for the next step, thereby avoiding limitations in deterministic prediction by GpyOpt and Gryffin.^35,36^ When the CO yield = 0, the objective function is equal to – ∞ (eq 1); which was replaced with −1000 for compatibility with the algorithms. Bounds were set for each of the five variables: Catalyst 1.5:10 μM, Photosensitizer 10:180 μM, Surfactant 1:40 CMC, Reductant 1:500 mM, Buffer 10:1000 mM. Where multiple suggested points were deemed comparable, they were skipped for the sake of maximum capitalization of the experimental setup.

### Machine Learning Regression and Feature Importance

Experimental data harvested during the experiments was used to train/validate five Random Forest regression-based pipelines with hyperparameter tuning in 5-fold cross-validation. Conditions with no CO, corresponding to *y*^obj 1^ = −1000, were deemed outliers and the same training/testing sets were used for all the models. Specifically, the space of hyperparameters is composed by all the possible combinations of (i) the number of estimators, to be chosen among [100, 200, 500, 1000, 2000] and (ii) the number of features to keep when looking for the best split, to be chosen among [’auto’,’sqrt’,’log2’]. For the database handling and for the models training/validation Pandas^46^ and the Scikit-Learn^47^ Python packages were respectively employed. The TreeSHAP algorithm^17,18^ was used to identify feature rankings over the corresponding models’ outputs as it is suitable for tree-based models like Random Forest.

### Features grouping

The feature grouping separates values into classes based on a threshold for the corresponding properties, i.e., above (class 1) and below (class 0) the threshold following the methodology recently introduced by Trezza & Chiavazzo.^20^ Such thresholds are −2.2 for *y*^obj 1^, 1.3 μmol for CO, 300 for TON_CO_, 18.5 min^–1^ for TOF_CO_, 0.14 for QY_CO_. In particular, given the original set of 5 features (*x*_1_, *..., x*_5_), the corresponding dimensionless quantities are denoted (*x̃*_1_, ..., *x̃*_5_), where  representing the minimum and the maximum value of the *i–*th feature over the training set, respectively. A new set of two mixed features (*x*_1_, *x*_2_) is thus defined, where *x*_*j*_ = ∏_*i* = 1_^5^*x̃*_*i*_^*α*_*ij*_^, with  being a matrix computed by means of a multiobjective optimization criterion in two steps. First, the matrix {*α*_*ij*_} is the utopia point of the Pareto front simultaneously attempting (i) the maximization of the distance between the two classes according to the Bhattacharyya distance,^48,49^ (ii) the minimization of a norm of the covariance matrix of the first class distribution, (iii) the minimization of a norm of the covariance matrix of the second class distribution. Second, the solution of the first optimization step is used as the input of a nearest neighbor optimization, to minimize the number of neighbors of class 0 to each sample of class 1 within a fixed cutoff radius of 10^–2^. Finally, the new variables normalized in the interval [0, 1] are computed as .

## Data Availability

The primary data supporting the findings of this study are available from the University of Cambridge open-access data repository (https://doi.org/10.17863/CAM.108306) with additional information available from the corresponding authors upon reasonable request.

## Supporting Information Available

Tabulated values from photocatalytic testing and learning algorithm optimization (in order of algorithm input/output) and transient absorption spectroscopy; supplementary notes elaborating on photoluminescence, regression model design and controlling feature grouping; transient absorption spectra; plots of photocatalytic results including ^13^C labeling, exclusion controls, measurements over time, tests with cationic and anionic surfactants; a more technical depiction of the learning algorithm optimization, correlation plot matrices and controlling feature grouping plots (PDF)
