# Supplementary material for: Multi-Variable Multi-Metric Optimization of Self-Assembled Photocatalytic CO2 Reduction Performance Using Machine Learning Algorithms
Source: J Am Chem Soc. 2024 May 20;146(22):15648–58. doi: 10.1021/jacs.4c01305 (PMC11157525; doi:10.1021/jacs.4c01305)
Supplement: Supplementary file 1 — ja4c01305_si_001.pdf [file ja4c01305_si_001.pdf]

Supplementary Information for

Multi-Variable Multi-Metric Optimization of Self-Assembled Photocatalytic CO_2 Reduction Performance using Machine Learning Algorithms

Shannon A. Bonke,[a] Giovanni Trezza,[b] Luca Bergamasco,[b] Hongwei Song,[c] Santiago Rodríguez-Jiménez,[a] Leif Hammarström,[c] Eliodoro Chiavazzo[b]* and Erwin Reisner.[a]*

10.1021/jacs.4c01305

[a] Yusuf Hamied Department of Chemistry, University of Cambridge, Lensfield Rd, Cambridge CB2 1EW, UK.

[b] Department of Energy, Politecnico di Torino, Corso Duca degli Abruzzi 24, 10129 Turin, Italy.

[c] Department of Chemistry, Ångström Laboratory, Uppsala University, Lägerhyddsvägen 1, 752 37 Uppsala, Sweden.

* Correspondence to: Prof E. Reisner (reisner@ch.cam.ac.uk) or Prof E. Chiavazzo (eliodoro.chiavazzo@polito.it).

Contents

Supplementary Notes	2
Supplementary Tables	5
Supplementary Figures	10

Supplementary Notes

1. Calculation Formulae for Metrics. The following figures of merit were quantified in photocatalytic tests:

Metric	Symbol	Units	Calculation
Yield of CO	$Yield_{CO}$	μmol h^{-1}	μmol(CO) / time
Turnover Number of Catalyst	TON_{CO}		μmol(CO) / μmol(catalyst)
Turnover Frequency of Catalyst	TOF_{CO}	**min^{-1}**	TON / time
Turnover Number of Photosensitizer (PS)	TON_{PS}		μmol(CO) / μmol(PS)
Turnover Frequency of Photosensitizer (PS)	TOF_{PS}	**min^{-1}**	TON(PS) / time
Quantum Yield of Reaction	QY_{CO}	%	μmol(CO) / μmol(photons) *100
Selectivity for CO	Sel_{CO}	%	μmol(CO) / (μmol(CO) + μmol(H_2)) *100

2. Photoluminescence (PL) and Transient Absorption Spectroscopy (TAS). Photoexcited non-alkylated Rubpy decays with a lifetime (τ) of 560 ns in water or 525 ns in phosphate buffer, with minimal effect from the presence of surfactant (Fig S5, Table S2). In contrast, Ru* of $Rubpy_{C17}$ shows a more rapid double-exponential decay with comparable lifetimes in water or phosphate buffer (τ_1 31 ns, 63%, τ_2 394 ns, 37%; Fig S5 and Table S2). This is attributed to self-quenching being faster for $Rubpy_{C17}$ assembled into micelles (higher local concentration) compared to freely diffusing Rubpy. Adding 3 CMC of $C_{12}E_6$ surfactant increases the Ru* lifetime for $Rubpy_{C17}$ (τ_1 50 ns, 62%, τ_2 503 ns, 38%; Fig S5, Table S2), with the effect attributed to the surfactant mitigating self-quenching by forming mixed $Rubpy_{C17}$/surfactant micelles that result in longer Ru-Ru distances (decrease in local concentration). The $Rubpy_{C17}$* lifetime shows quencher concentration dependence, thereby excluding the possibility of static quenching by ascorbate electrostatically assembled at the charged micelle surface (Fig S6, Table S3).

In the presence of the reductive quencher, NaAsc, the absorption peak at 510 nm enables quantification of Ru^- and determination of its lifetime. The intensity of this peak is unchanged for Rubpy in water, phosphate buffer and $C_{12}E_6$ surfactant (40 mOD), while $Rubpy_{C17}$ shows ~50% less Ru^- forming in water and buffered media (17 mOD; Fig S7 and Table S4). This is expected as most Ru* is decaying through self-quenching before it can be quenched to Ru^- by ascorbate. The inclusion of $C_{12}E_6$ surfactant increases the reduced $Rubpy_{C17}$ concentration to 75% of the diffusionally free Rubpy value (30 vs 40 mOD), most probably due to the longer Ru* lifetime enabling more centers to be quenched.

Both Rubpy and $Rubpy_{C17}$ show single exponential decay of Ru^- (Fig S8, Table S5), yet the Rubpy lifetime is below 100 μs in water, phosphate buffer and $C_{12}E_6$ solution (72, 62 and 16 μs), whereas $Rubpy_{C17}$ shows a lifetime >200 μs under these conditions (247, 216 and 282 μs). These lifetimes are further increased when catalyst is added into the micelles (without changes to Ru^- yield), with τ for $Rubpy_{C17}/C_{12}E_6$ micelles increasing 33% (282 to 375 μs) while Rubpy shows no change (Fig S8, Table S5). This leads to a 23-fold longer Ru^- lifetime for $Rubpy_{C17}$ in $C_{12}E_6$ micelles with catalyst compared to the standard Rubpy (375 vs 16 μs). The $Rubpy_{C17}^-$ lifetime at 510 nm was not sensitive to catalyst loading from 1.5 to 5 μM (375 ± 13 vs 370 ± 6 μs; Fig S11A), nor sensitive to switching from Ar to CO_2 saturated media (Fig S11B). Charge separated states from direct photoexcitation of the catalyst were not observable on the ns-timescale employed.

Contrasting the $Rubpy_{C17}/C_{12}E_6$ system against the catalytically inactive cationic and anionic surfactants shows that the photoexcited Ru* lifetime is higher in cationic CTAC (note chloride salt used rather than bromide), while SDS prevents self-quenching as effectively as using freely diffusing Rubpy (Fig S5, Table S2). Cationic CTAC (note chloride salt used rather than bromide, so bromide quenching cannot be excluded) resulted in $Rubpy_{C17}$ showing a double-exponential decay like $C_{12}E_6$, but the weightings for the

two processes were inverted to give more Ru* a longer lifetime (τ_1 67 ns, 28%, τ_2 585 ns, 72% for CTAC compared to τ_1 31 ns, 63%, τ_2 394 ns, 37% for $C_{12}E_6$). This trend extended with $Rubpy_{C17}$/SDS, where only the slower decay pathway of Ru* was observed and the lifetime was comparable to freely diffusing Rubpy (τ 684 ns for $Rubpy_{C17}$/SDS vs 667 ns for Rubpy in SDS solution). This indicates that SDS very effectively prevents self-quenching of Ru* in the micelles. The $Rubpy_{C17}$* lifetime was dependent on the NaHAsc concentration in CTAC, but independent in SDS as there was no lifetime change compared to freely diffusing Rubpy absent NaAsc (Fig S6).

The yield of $Rubpy_{C17}^-$ was 17% lower with CTAC than $C_{12}E_6$ (25 vs 30 mOD; Fig S7), whereas negligible $Rubpy_{C17}^-$ was formed in SDS micelles (4 mOD) presumably due to electrostatic repulsion between the negative charges of surfactant and reductant preventing formation of a 'solvent cage' to explain their catalytic inactivity. The $Rubpy_{C17}^-$ lifetime in $C_{12}E_6$ micelles is 2.4-fold higher than CTAC (282 vs 119 µs), which may explain the lack of catalytic turnover with electron transfer to the catalyst being limiting.

3. Calculation of Number of Points in Parameter Space (Simplex Size). The concentrations of each variable are continuous variables that must be discretized a fixed number of points. Testing 10 different concentrations of each variable would give reasonable resolution of performance peaks. With five different solutions, each tested at 10 concentrations in all combinations, this is 10^5 combinations.

4. Cross-validation of Regression Model. The widely recognized *k*-fold cross-validation approach was employed to identify the most effective hyperparameters for the regression models, with *k*=5. The dataset of 103 samples, each containing 5 features along with the target property to be predicted, was divided into a training set (72 samples) and a testing set (31 samples). The training set is further divided into five subsets or folds. All possible combinations of (i) the number of estimators, selected from [100, 200, 500, 1000, 2000], and (ii) the number of features to retain for the optimal split, chosen from ['auto', 'sqrt', 'log2'], were examined and resulted in 15 distinct hyperparameter combinations. For each hyperparameter combination, the model underwent training on four folds while its performance was assessed on the fifth fold, serving as the validation set. This cross-validation process was repeated five times, with each iteration using a different fold as the validation data and the remaining four as training. Following this, the average performance across all five validation folds was calculated for each hyperparameter combination, and the best-performing combination was chosen. Using the top-performing hyperparameter combination, the final model was trained on all five folds, and the trained model was then utilized to predict the target property on the testing set.

5. Control Group Feature Analysis. The maximum performance is on a peak which appears particularly sensitive to small changes, but alternative parameter combinations can also be shown for more stable regions such as the fourth best performance combination, where an alternative combination of parameters can be found that also lowers the catalyst loading by 30 % while requiring 7 % less photosensitizer (Catalyst 2.7 vs. 3.8 µM, Photosensitizer 88 vs 95 µM, Surfactant 17 vs 14 CMC, Reductant 162 vs 136 mM, Buffer 262 vs 305 mM). Implementation details are shown below.

To find points with the same performances and different concentrations of the original features $x_1, \dots, x_5$, we have solved the following system of two implicit equations:

$$\left(\frac{\eta_1 x_1 - m_1}{M_1 - m_1} + 1\right)^{\alpha_{11}} \left(\frac{\eta_2 x_2 - m_2}{M_2 - m_2} + 1\right)^{\alpha_{21}} \left(\frac{\eta_3 x_3 - m_3}{M_3 - m_3} + 1\right)^{\alpha_{31}} \left(\frac{\eta_4 x_4 - m_4}{M_4 - m_4} + 1\right)^{\alpha_{41}} \left(\frac{\eta_5 x_5 - m_5}{M_5 - m_5} + 1\right)^{\alpha_{51}} = \mathrm{const}_1 \times \left(\widehat{M_1} - \widehat{m_1}\right) + \widehat{m_1}$$

$$\left(\frac{\eta_1 x_1 - m_1}{M_1 - m_1} + 1\right)^{\alpha_{12}} \left(\frac{\eta_2 x_2 - m_2}{M_2 - m_2} + 1\right)^{\alpha_{22}} \left(\frac{\eta_3 x_3 - m_3}{M_3 - m_3} + 1\right)^{\alpha_{32}} \left(\frac{\eta_4 x_4 - m_4}{M_4 - m_4} + 1\right)^{\alpha_{42}} \left(\frac{\eta_5 x_5 - m_5}{M_5 - m_5} + 1\right)^{\alpha_{52}} = = \mathrm{const}_2 \times \left(\widehat{M_2} - \widehat{m_2}\right) + \widehat{m_2}$$

where M_1, M_2, M_3, M_4, M_5 are the maximum values observed in the training set for Catalyst, Photosensitiser, Surfactant, Reductant, Buffer concentrations respectively, m_1, m_2, m_3, m_4, m_5 are the minimum values observed in the training set for Catalyst, Photosensitiser, Surfactant, Reductant, Buffer concentrations respectively, x_1, x_2, x_3, x_4, x_5 denote the Catalyst, Photosensitiser, Surfactant, Reductant, Buffer concentrations respectively (Table S9), $\alpha_{ij}s$ represent the exponents to construct the optimised features for objective function 1 (namely, the first two rows in Table S10), const_1 and const_2 are the first and second coordinates in the optimised features chart of the point we want to find an alternative combination for (namely, $x_{1,\mathrm{obj\ 1,norm}}$ and $x_{2,\mathrm{obj\ 1,norm}}$ in Fig. S13), $\widehat{M_1}, \widehat{M_2}, \widehat{m_1}, \widehat{m_2}$ are the maximum and minimum values observed over the training set for the non-normalised mixed features referred to objective function 1 (namely, the first two rows in Table S11).

We aimed at finding the values of the five coefficients $\eta_2, \dots, \eta_5$ such that those two equations were solved within an error of 1e-4 on the value of the const, imposing $\eta_1 = 0.7$ and $\eta_2 < 1$.

In particular, for the best point they become

$$\left(\frac{3.9\,\eta_1 - 1.5}{8.6 - 1.5} + 1\right)^{0.216} \left(\frac{112.4\,\eta_2 - 2.6}{168 - 2.6} + 1\right)^{0.150} \left(\frac{19.0\,\eta_3 - 10}{33.7 - 10} + 1\right)^{0.207} \left(\frac{219.6\,\eta_4 - 1}{500 - 1} + 1\right)^{-0.408} \times \left(\frac{461.5\,\eta_5 - 72}{870 - 72} + 1\right)^{-0.850} = 0.447 \times (0.998 - 0.587) + 0.587$$

$$\left(\frac{3.9\,\eta_1 - 1.5}{8.6 - 1.5} + 1\right)^{0.083} \left(\frac{112.4\,\eta_2 - 2.6}{168 - 2.6} + 1\right)^{0.073} \left(\frac{19.0\,\eta_3 - 10}{33.7 - 10} + 1\right)^{-0.041} \left(\frac{219.6\,\eta_4 - 1}{500 - 1} + 1\right)^{-0.509} \times \left(\frac{461.5\,\eta_5 - 72}{870 - 72} + 1\right)^{-0.852} = 0.303 \times (0.998 - 0.587) + 0.587$$

while for the 4th best point it becomes

$$\left(\frac{3.8\,\eta_1 - 1.5}{8.6 - 1.5} + 1\right)^{0.216} \left(\frac{94.6\,\eta_2 - 2.6}{168 - 2.6} + 1\right)^{0.150} \left(\frac{14.3\,\eta_3 - 10}{33.7 - 10} + 1\right)^{0.207} \left(\frac{135.5\,\eta_4 - 1}{500 - 1} + 1\right)^{-0.408} \times \left(\frac{305.1\,\eta_5 - 72}{870 - 72} + 1\right)^{-0.850} = 0.721 \times (0.998 - 0.587) + 0.587$$

$$\left(\frac{3.8\,\eta_1 - 1.5}{8.6 - 1.5} + 1\right)^{0.083} \left(\frac{94.6\,\eta_2 - 2.6}{168 - 2.6} + 1\right)^{0.073} \left(\frac{14.3\,\eta_3 - 10}{33.7 - 10} + 1\right)^{-0.041} \left(\frac{135.5\,\eta_4 - 1}{500 - 1} + 1\right)^{-0.509} \times \left(\frac{305.1\,\eta_5 - 72}{870 - 72} + 1\right)^{-0.852} = 0.541 \times (0.998 - 0.587) + 0.587$$

Given $\eta_1 = 0.7$ (coefficient of the Catalyst), we have explored all the possible combinations of $\eta_2, \dots, \eta_5$ from 0.7 to 1.2 and with a step of 0.005; the two proposed combinations are the ones minimizing η_2 (coefficient of the Photosensitiser).

Supplementary Tables

Table S1. Tabulated values from initial experiments, exclusion controls and kinetic study.[a]

#	Cat	PS	Surf.	Red.	Buf.	CO / µmol			TON_{CO}		TOF_{CO} / min^{-1}		Quant. Yield		Sel CO:H2		A456 nm		
	µM	µM	CMC	mM	mM	Mean	±SD	%RSD	Mean	±SD	Mean	±SD	Mean	±SD	Mean	±SD	0 min	15 min	Post/Pre
**All**[b]	1.5	30	3	100	100	0.37	0.06	17	249	38	17	2	0.04	0.01	87	2	0.39±0.03	0.27±0.04	0.70
**No Surf.**	1.5	30	**0**	100	100	0.077	0.002	3	52	2	3.4	0.1	0.0082	0.0003	84	3	0.38	0.31	0.70
**N_2**[c]	1.5	30	3	100	100	0.001	0.002	-	1	1	0.04	0	0.0	0.0	2	3	0.43	0.37	0.86
**No Cat**	0.0	30	3	100	100	0.001	0.001	-	-	-	0.04	0	0.0	0.0	40	53	0.35	0.43	1.23
**No PS**	1.5	**0**	3	100	100	0	-	-	0	-	-	-	0	-	-	-	-	0.07	-
**No Red.**	1.5	30	3	**0**	100	0	-	-	0	-	-	-	0	-	-	-	0.43	0.40	0.92
**Dark**[d]	1.5	30	3	100	100	0	-	-	0	-	-	-	0	-	-	-	0.43	0.41	0.95
**Rubpy**[e]	1.5	30	3	100	100	0.15	0.02	13	102	12	6.80	0.80	0.016	0.002	58	0	0.42	0.25	0.60
**15 min**	1.5	30	3	100	100	0.38	0.02	5	254	14	16.9	1	0.04	0.002	89	0	0.41	0.30	0.73
**30min**	1.5	30	3	100	100	0.52	0.08	15	350	56	12	2	0.028	0.004	88	0	0.41	0.25	0.61
**45 min**	1.5	30	3	100	100	0.7	0	0	494	0	11	0	0.03	0	85	0	0.41	0.22	0.54
**60 min**	1.5	30	3	100	100	0.8	0.06	8	531	42	8.8	0.7	0.021	0.002	86	0	0.41	0.20	0.48
**75 min**	1.5	30	3	100	100	0.9	0	0	585	0	8	0	0.02	0	85	0	0.41	0.19	0.46
**90 min**	1.5	30	3	100	100	0.85	0.01	1	569	10	6.3	0.1	0.015	0.0003	80	1	0.41	0.20	0.48
**105 min**	1.5	30	3	100	100	1.0	0	0	650	0	6	0	0.01	0	80	0	0.41	0.18	0.43
**120 min**	1.5	30	3	100	100	0.9	0.1	11	600	69	5	0.6	0.012	0.001	79	2	0.41	0.19	0.47

[a] Solutions CO_2-sat. (pH 6.3) and illuminated for 15 min with 447 nm illumination with 2.3 W LED at 25 °C with 250 rpm orbital shaking, 1 mL reaction volume. All errors are ± 1 standard deviation. Cat = $CoPyP_{C16}$, PS = $Rubpy_{C17}$, Surf. = $C_{12}E_6$, Red. = NaHAsc, Buf. = phosphate buffer. [b] Batch-to-batch average for 8 batches of 2-3 replicates conducted over ~1 year. [c] Solution purged with N_2 to exclude CO_2 (pH 7.0). [d] Solution kept in dark. [e] $[Ru(bpy)_3]Cl_2$ as photosensitizer in place of $Rubpy_{C17}$.

Table S2: Self-quenching rates (τ / ns) and relative contribution for double-exponential processes for photosensitizers obtained from photoluminescence decay (pump 460 nm, probe 620 nm) of Rubpy and $Rubpy_{C17}$ absent ascorbate quencher. 30 µM photosensitizer in Ar purged aqueous media; phosphate buffer (0.1 M, pH 7.0) and/or surfactant at 3 CMC if present.

	Rubpy	$Rubpy_{C17}$	
Water	560	29<br>59%	470<br>41%
Phosphate Buffer	525	31<br>63%	394<br>37%
$C_{12}E_6$ in PB	503	50<br>62%	503<br>38%
CTAC in PB	521	67<br>28%	585<br>72%
SDS in PB	667	-	684<br>100%

Table S3: Quenching rates (τ / µs) for photosensitizers obtained from transient absorption spectroscopy (pump 460 nm, probe 620 nm) of $Rubpy_{C17}$ with ascorbate quencher. 30 µM photosensitizer in phosphate buffer (0.1 M, pH 7.0), 100 mM sodium ascorbate, Ar purged; surfactant at 3 CMC.

	$Rubpy_{C17}$				
**[NaAsc]**	***$C_{12}E_6$***		***CTAC***		***SDS***
**0.1 mM**	50<br>59%	482<br>41%	59<br>33%	529<br>67%	644<br>100%
**1 mM**	40<br>56%	344<br>44%	46<br>23%	277<br>77%	723<br>100%
**10 mM**	15<br>55%	91<br>45%	47<br>100%	-	766<br>100%
**100 mM**	10<br>100%	-	11<br>100%	-	692<br>100%

Table S4: Initial intensity of peak at 510 nm from transient absorption spectroscopy (pump 460 nm, probe 510 nm) of Rubpy and Rubpy$_{C17}$ with ascorbate quencher. 30 µM photosensitizer in phosphate buffer (0.1 M, pH 7.0), 100 mM sodium ascorbate, Ar purged; surfactant at 3 CMC if present.

	Intensity at 510 nm / mOD	
	Rubpy	Rubpy$_{C17}$
Water	40	17
Phosphate Buffer	40	16
$C_{12}E_6$ in PB	40	30
CTAC in PB	40	25
SDS in PB	15	4

Table S5: Reductively quenched photosensitizer decay rates (τ / µs) obtained from transient absorption spectroscopy (pump 460 nm, probe 510 nm) of Rubpy and Rubpy$_{C17}$ with ascorbate quencher. 30 µM photosensitizer in phosphate buffer (0.1 M, pH 7.0), 100 mM sodium ascorbate, Ar purged; surfactant at 3 CMC and catalyst at 1.5 µM if present.

	Rubpy		Rubpy$_{C17}$	
	No Cat	Cat	No Cat	Cat
Water	72	50	247	252
Phos. Buf.	62	26	216	414
$C_{12}E_6$ in PB	16	16	282	375
CTAC in PB	25	11	119	140
SDS in PB	533	604	N/A	N/A

Table S6. Tabulated values from heuristic optimization.[a]

	Cat	PS	Surf.	Red.	Buf	CO / µmol			TON$_{CO}$		TOF$_{CO}$ / min^{-1}		Quant. Yield		Sel CO:H2		A456 nm		
#	µM	µM	CMC	mM	mM	Mean	±SD	%RSD	Mean	±SD	Mean	±SD	Mean	±SD	Mean	±SD	0 min	15 min	Post/Pre
1	1.5	30	3	100	100	0.37	0.06	17	249	38	17	2	0.04	0.01	87	2	0.385±0.03	0.27±0.04	0.70
2*	**0.01**	30	3	100	100	0.051	0.005	10	5142	469	86	8	0.0014	0.0001	25	2	0.34	0.17	0.50
3*	**0.1**	30	3	100	100	0.07	0.01	14	661	128	11	2	0.0017	0.0003	50	18	0.35	0.21	0.59
4*	**0.5**	30	3	100	100	0.34	0.05	15	688	95	11	2	0.009	0.001	78	5	0.36	0.21	0.58
5*	**1**	30	3	100	100	0.58	0.01	2	578	13	9.6	0.2	0.0153	0.0003	84	0	0.38	0.20	0.52
6*	**1.5**	30	3	100	100	0.7	0.1	14	472	71	8	1	0.019	0.003	86	1	0.40	0.21	0.52
7*	**3**	30	3	100	100	1.14	0.08	7	379	28	6.3	0.5	0.03	0.002	88	0	0.47	0.22	0.46
8*	**5**	30	3	100	100	1.33	0.1	8	266	20	4.4	0.3	0.035	0.003	88	1	0.55	0.24	0.44
9*	**10**	30	3	100	100	1.51	0.06	4	151	6	2.51	0.1	0.04	0.002	89	0	0.78	0.40	0.52
10	1.5	**3**	3	100	100	0.04	0.02	50	30	10	2	0.7	0.005	0.002	87	3	0.09	0.03	0.33
11	1.5	**10**	3	100	100	0.18	0.02	11	119	17	8	1	0.019	0.003	88	1	0.16	0.08	0.50
12	1.5	**50**	3	100	100	0.37	0.03	8	243	20	16	1	0.039	0.003	89	1	0.58	0.45	0.78
13	1.5	**100**	3	100	100	0.218	0.008	4	146	5	9.7	0.3	0.0231	0.0008	89	0	1.05	1.04	0.99
14	1.5	**200**	3	100	100	0.077	0.004	5	51	3	3.4	0.2	0.0082	0.0005	88	1	1.66	1.82	1.10
15	1.5	30	**1**	100	100	0.2	0.01	5	131	9	8.7	0.6	0.021	0.001	90	1	0.40	0.29	0.73
16	1.5	30	**6**	100	100	0.27	0.04	15	184	28	12	2	0.029	0.004	88	1	0.41	0.30	0.73
17	1.5	30	**12**	100	100	0.28	0.05	18	189	31	13	2	0.03	0.005	89	0	0.42	0.32	0.76
18	1.5	30	**18**	100	100	0.28	0.05	18	189	31	13	2	0.03	0.005	90	1	0.42	0.35	0.83
19	1.5	30	3	**1**	100	0.013	0.002	15	9	1	0.6	0.09	0.0014	0.0002	95	1	0.41	0.34	0.83
20	1.5	30	3	**10**	100	0.157	0.005	3	105	4	7	0.2	0.0166	0.0006	88	0	0.39	0.29	0.74
21	1.5	30	3	**500**	100	0.25	0.02	8	170	11	11.3	0.8	0.027	0.002	83	0	0.35	0.20	0.57
22	1.5	30	3	**1000**	100	0.25	0.08	32	170	52	11	3	0.027	0.008	82	4	0.35	0.19	0.54
23	1.5	30	3	100	**1**	0.29	0.02	7	193	11	12.8	0.7	0.031	0.002	85	1	0.39	0.24	0.62
24	1.5	30	3	100	**10**	0.3	0.06	20	198	39	13	3	0.031	0.006	87	0	0.40	0.25	0.63
25	1.5	30	3	100	**500**	0.27	0.01	4	178	7	11.9	0.5	0.028	0.001	89	1	0.38	0.22	0.58
26	1.5	30	3	100	**1000**	0.09	0.1	111	58	65	4	4	0.01	0.01	88	2	0.65	0.17	0.26

[a] Solutions CO_2-sat. (pH 6.3) and illuminated for 15 min with 447 nm illumination with 2.3 W LED at 25 °C with 250 rpm orbital shaking, 1 mL reaction volume. Cat = CoPyP$_{C16}$, PS = Rubpy$_{C17}$, Surf. = $C_{12}E_6$, Red. = NaHAsc, Buf. = phosphate buffer. * 60 min reaction.

Table S7. Tabulated values from learning algorithm optimization.[a]

	Cat/	PS	Surf.	Red.	Buf	CO / µmol			TON_{CO}		TOF_{CO} / min^{-1}		Quant. Yield		Sel CO:H2		A456 nm		
#	µM	µM	CMC	mM	mM	Mean	±SD	%RSD	Mean	±SD	Mean	±SD	Mean	±SD	Mean	±SD	0 min	15 min	Post/Pre
1*	1.5	30	3	100	100	0.37	0.06	17	249	38	17	2	0.04	0.01	87	2	0.385±0.03	0.27± 0.04	0.70
2*	1.5	**10**	3	100	100	0.18	0.02	11	119	17	8	1	0.019	0.003	88	1	0.16	0.08	0.50
3*	1.5	**50**	3	100	100	0.37	0.03	8	243	20	16	1	0.039	0.003	89	1	0.58	0.45	0.78
4*	1.5	**100**	3	100	100	0.218	0.008	4	146	5	9.7	0.3	0.0231	0.0008	89	0	1.05	1.04	0.99
5*	1.5	30	**1**	100	100	0.2	0.01	5	131	9	8.7	0.6	0.021	0.001	90	1	0.40	0.29	0.73
6*	1.5	30	**6**	100	100	0.27	0.04	15	184	28	12	2	0.029	0.004	88	1	0.41	0.30	0.73
7*	1.5	30	**12**	100	100	0.28	0.05	18	189	31	13	2	0.03	0.005	89	0	0.42	0.32	0.76
8*	1.5	30	**18**	100	100	0.28	0.05	18	189	31	13	2	0.03	0.005	90	1	0.42	0.35	0.83
9	5.1	87	17	177	460	0.6	0.03	5	118	6	7.8	0.4	0.063	0.003	88	0	1.36	1.02	0.75
10	3.6	115	30	242	252	1.07	0.06	6	297	16	20	1	0.113	0.006	89	2	1.53	1.12	0.73
11	4.7	112	17	242	445	0.5	0.08	16	107	17	7	1	0.053	0.008	83	2	1.54	1.02	0.66
12	1.5	30	3	96	72	0	0.009	3	197	6	13.1	0.4	0	0.001	89	1	0.37	0.28	0.76
13	5.8	95	30	174	584	0	0	0	0	0	0	0	0	0	0	0	-	-	-
14	7.2	96	17	198	598	0.18	0.06	33	25	9	1.6	0.6	0.019	0.007	86	1	0.45	0.44	0.98
15	3.0	135	22	177	613	0.27	0.00	0	89	0	5.9	0	0.03	0.00	89	0	0.63	0.34	0.54
16	3.2	99	26	193	593	0.297	0.002	1	93	1	6.17	0.05	0.0314	0.0002	89	1	0.37	0.47	1.27
17	2.2	47	8	109	132	0.54	0.03	6	246	12	16.4	0.8	0.057	0.003	88	0	0.58	0.40	0.69
18	6.2	75	30	195	638	0.34	0.01	3	55	2	3.6	0.1	0.036	0.001	88	1	0.35	0.45	1.29
19**	1.5	30	3	**1**	100	0.013	0.002	15	9	1	0.6	0.09	0.0014	0.0002	95	1	0.41	0.34	0.83
20**	1.5	30	3	**10**	100	0.157	0.005	3	105	4	7	0.2	0.0166	0.0006	88	0	0.39	0.29	0.74
21**	1.5	30	3	**500**	100	0.25	0.02	8	170	11	11.3	0.8	0.027	0.002	83	0	0.35	0.20	0.57
22**	1.5	30	3	100	**10**	0.3	0.06	20	198	39	13	3	0.031	0.006	87	0	0.40	0.25	0.63
23**	1.5	30	3	100	**500**	0.27	0.01	4	178	7	11.9	0.5	0.028	0.001	89	1	0.38	0.22	0.58
24**	1.5	30	3	100	**1000**	0.09	0.1	111	58	65	4	4	0.01	0.01	88	2	0.65	0.17	0.26
25	1.5	104	29	204	596	0.249	0.007	3	166	4	11.1	0.3	0.0263	0.0007	90	0	1.86	0.44	0.24
26	2.8	85	21	175	491	0.363	0.007	2	130	2	8.6	0.1	0.0384	0.0007	89	1	1.37	0.56	0.41
27	5.5	117	29	260	383	0.7	0.08	11	127	16	8	1	0.074	0.009	92	1	1.84	1.24	0.67
28	7.5	97	28	268	731	0.27	0.01	4	36	2	2.4	0.1	0.028	0.001	90	1	1.73	0.21	0.12
29	3.1	99	26	194	593	0.32	0.01	3	103	4	6.8	0.2	0.034	0.001	91	0	1.88	0.54	0.29
30	5.5	117	26	186	611	0.34	0.04	12	63	8	4.2	0.5	0.036	0.004	91	0	1.93	0.55	0.28
31	7.0	78	26	344	673	0.16	0.08	50	23	13	1.5	0.8	0.017	0.009	89	2	1.51	0.17	0.11
32	4.8	69	21	152	312	0.52	0.04	8	109	8	7.2	0.5	0.055	0.004	86	1	1.22	0.69	0.57
33	3.3	113	34	190	513	0.4	0.02	5	122	8	8.1	0.5	0.042	0.003	83	0	1.38	0.65	0.47
34	2.7	77	18	155	383	0.46	0.01	2	169	4	11.3	0.3	0.048	0.001	85	0	1.20	0.69	0.58
35	7.3	107	17	251	641	0.3649	0.0006	0	50	0	3	0	0.03861	7E-05	85	1	1.59	0.46	0.29
36	5.5	144	30	291	600	0.38	0.02	5	69	4	4.6	0.2	0.04	0.002	84	1	1.36	0.66	0.48
37	2.5	92	23	175	478	0.39	0.02	5	86	5	5.7	0.3	0.041	0.002	85	0	1.55	0.48	0.31
38	6.4	125	25	249	561	0.52	0.03	6	156	8	10.4	0.6	0.055	0.003	86	1	1.91	0.43	0.22
39	6.5	99	27	274	808	0.14	0.09	64	55	35	4	2	0.014	0.009	80	4	1.52	0.12	0.08
40***	4.5	90	9	100	100	0.8	0.2	25	175	38	12	3	0.08	0.02	84	0	1.03	0.70	0.68
41***	4.5	90	9	500	100	1.06	0.08	8	236	17	16	1	0.113	0.008	82	0	1.08	0.69	0.64
42***	6	120	12	100	100	1.06	0.02	2	178	2	11.8	0.1	0.113	0.002	86	1	1.36	1.10	0.81
43***	6	120	12	500	100	1.37	0.06	4	229	10	15.3	0.7	0.145	0.006	85	4	1.461	0.999	0.68
44***	9	180	18	500	100	1.64	0.03	2	183	4	12.2	0.2	0.174	0.003	85	0	2.175	1.628	0.75
45	3.4	104	31	189	503	1.1	0.03	3	323	9	21.5	0.6	0.116	0.003	89	0	1.32	1.03	0.78
46	2.2	109	32	195	509	0.93	0.02	2	422	8	28.1	0.6	0.098	0.002	89	0	1.35	1.12	0.83
47	3.8	95	14	136	305	1.32	0.05	4	349	13	23.2	0.9	0.14	0.005	89	0	1.29	0.95	0.73
48	6.1	141	26	392	399	1.65	0.02	1	271	4	18	0.2	0.174	0.002	88	1	1.86	1.41	0.76
49	6.4	53	19	285	725	0.29	0.04	14	46	6	3	0.4	0.031	0.004	89	0	1.16	0.20	0.17
50	2.4	94	31	168	396	1.0	0.2	20	415	66	28	4	0.11	0.02	91	1	1.21	0.91	0.75
51	4.2	79	11	138	303	1.24	0.05	4	295	11	19.7	0.8	0.131	0.005	90	1	1.14	0.75	0.66
52	5.5	118	23	354	575	0.74	0.01	1	134	3	8.9	0.2	0.078	0.001	90	1	1.42	1.22	0.86

**53**	7.0	77	30	346	573	0.72	0.04	6	103	6	6.8	0.4	0.076	0.005	90	0	1.13	0.88	0.78
**54**	2.9	100	31	189	502	0.9	0.1	11	320	35	21	2	0.1	0.01	89	0	1.25	0.93	0.75
**55**	2.3	113	36	190	510	0.74	0.06	8	323	24	22	2	0.079	0.006	89	0	1.35	1.16	0.86
**56**	5.4	107	21	216	462	1.528	0.001	0	283	0	19	0	0.1617	0.0001	90	1	1.40	1.04	0.74
**57**	5.0	105	18	175	318	1.35	0.09	7	270	19	18	1	0.14	0.01	90	1	1.35	1.03	0.76
**58**	7.2	131	35	317	525	0.87	0.04	5	121	6	8.1	0.4	0.092	0.005	90	1	1.70	1.44	0.85
**59**	3.4	108	33	184	505	1.07	0.02	2	315	7	21	0.5	0.113	0.003	91	1	1.34	1.07	0.80
**60**	3.6	99	25	180	493	1.195	0.002	0	332	0	22.1	0	0.1265	0.0002	90	0	1.26	0.95	0.75
**61**	4.5	88	25	243	305	1.3	0.1	8	284	25	19	2	0.14	0.01	91	1	1.16	0.85	0.73
**62**	8.0	147	25	342	584	0.703	0.007	1	88	1	5.83	0.05	0.0744	0.0007	89	1	1.91	1.62	0.85
**63**	2.1	119	38	209	607	0.35	0.01	3	168	7	11.2	0.5	0.037	0.002	87	1	1.39	1.43	1.03
**64**	3.6	117	34	211	584	0.6	0.01	2	167	4	11.1	0.3	0.064	0.001	88	0	1.45	1.29	0.89
**65**	3.1	96	24	174	484	1.08	0.03	3	350	9	23	0.6	0.115	0.003	88	0	1.20	0.88	0.74
**66**	6.2	132	22	260	489	1.4871	0.0002	0	240	0	16	0	0.15737	2E-05	87	1	1.71	1.31	0.77
**67**	9.8	98	25	348	323	1.8	0.08	4	184	8	12.2	0.5	0.19	0.008	87	1	1.47	0.95	0.65
**68**	2.9	100	28	177	442	0.99	0.02	2	343	8	22.8	0.5	0.105	0.002	88	1	1.24	0.949	0.77
**69**	4.2	104	20	168	397	1.17	0.06	5	280	15	18.6	1	0.124	0.006	89	1	1.33	0.918	0.69
**70**	8.6	122	24	287	494	1.52	0.02	1	176	3	11.7	0.2	0.16	0.003	88	1	1.66	1.14	0.68
**71**	8.9	104	19	193	525	1.56	0.05	3	176	5	11.7	0.3	0.165	0.005	89	0	1.45	1.01	0.70
**72**	7.6	104	28	234	562	0.99	0.05	5	131	7	8.7	0.5	0.105	0.006	89	0	1.41	1.12	0.80
**73**	3.0	85	17	144	318	1.112	0.004	0	371	1	25	0.09	0.1177	0.0004	90	1	1.07	0.80	0.75
**74**	6.4	128	18	409	690	0.5	0.03	6	79	4	5.3	0.3	0.053	3E-03	88	1	1.37	0.99	0.72
**75**	5.4	133	13	135	414	1.47	0.05	3	273	8	18.2	0.6	0.156	0.005	89	1	1.63	1.28	0.79
**76**	4.1	96	27	193	479	1.25	0.07	6	304	17	20	1	0.132	0.007	90	0	1.25	0.949	0.76
**77**	3.2	97	24	175	448	1.21	0.01	1	378	4	25.2	0.3	0.128	0.002	91	0	1.22	0.961	0.79
**78**	5.5	105	21	186	479	1.58	0.04	3	287	7	19.1	0.5	0.167	0.004	89	1	1.40	1.00	0.71
**79**	6.8	104	22	187	481	1.7	0.1	6	257	20	17	1	0.18	0.01	89	1	1.44	1.01	0.70
**80**	2.9	104	28	190	538	0.76	0.02	3	262	6	18	0.4	0.08	0.002	89	1	1.30	1.11	0.86
**81**	3.9	112	19	220	462	1.43	0.03	2	368	8	24.5	0.5	0.152	3E-03	88	0	1.43	1.11	0.78
**82**	4.7	159	10	214	734	1.04	0.02	2	221	4	14.7	0.2	0.11	0.002	87	0	1.90	1.58	0.83
**83**	9.4	103	23	224	611	0.63	0.02	3	68	2	4.5	0.1	0.067	0.002	89	0	1.304	0.897	0.69
**84**	4.1	107	24	187	519	1.13	0.009	1	276	2	18.4	0.1	0.1196	0.0009	89	0	1.39	1.136	0.82
**85**	5.6	130	11	54	442	1.57	0.05	3	280	8	18.7	0.6	0.166	0.005	89	0	1.627	1.33	0.82
**86**	7.6	108	14	181	541	1.482	0.005	0	195	1	12.97	0.05	0.1568	0.0005	88	1	1.50	1.10	0.73
**87**	7.4	103	9	178	545	1.4	0.04	3	189	6	12.6	0.4	0.148	0.004	88	1	1.43	1.07	0.75
**88**	5.1	105	22	185	546	1.01	0.05	5	199	10	13	0.7	0.107	0.005	89	0	1.42	1.17	0.82
**89**	6.2	139	13	404	698	0.42	0.02	5	68	3	4.5	0.2	0.045	2E-03	86	1	1.48	1.06	0.71
**90**	6.3	73	17	289	870	0.09	0.03	33	15	4	1	0.3	0.01	0.003	80	2	1.52	0.17	0.11
**91**	8.3	104	10	207	619	1.01	0.02	2	121	3	8.1	0.2	0.106	0.002	88	0	1.477	1.042	0.71
**92**	3.2	105	28	193	547	0.82	0.02	2	255	7	17.0	0.5	0.086	0.002	89	1	1.38	1.187	0.86
**93**	5.7	126	18	251	470	1.5739	0.0007	0	276	0	18.4	0	0.16655	7E-05	88	1	1.709	1.323	0.77
**94**	5.5	108	18	207	601	0.6	0.03	5	109	6	7.3	0.4	0.063	0.003	87	1	1.34	1.19	0.89
**95**	4.3	115	14	224	558	0.875	0.001	0	204	1	13.57	0.05	0.0926	0.0002	86	0	1.39	1.15	0.83
**96**	4.2	106	25	190	556	0.68	0.02	3	162	6	11	0.4	0.072	0.002	88	0	1.34	1.18	0.88
**97**	5.5	101	19	259	578	0.566	0.005	1	103	1	6.87	0.09	0.0599	5E-04	87	0	1.23	1.09	0.89
**98**	5.8	168	13	375	763	0.32	0.04	13	56	8	3.7	0.5	0.034	0.005	85	1	1.59	0.83	0.52
**99**	4.7	118	16	200	597	0.72	0.04	6	154	7	10.3	0.5	0.077	0.004	88	1	1.435	1.258	0.88
**100**	4.7	102	21	172	482	1.26	0.04	3	269	9	17.9	0.6	0.133	0.005	87	1	1.316	1.018	0.77
**101**	3.2	100	23	176	484	0.921	0.002	0	288	1	19.2	0.05	0.0974	0.0002	88	1	1.23	1.014	0.82
**102**	6.2	139	13	404	698	0.26	0.02	8	42	3	2.8	0.2	0.027	2E-03	83	1	1.354	0.353	0.26
**103**	6.3	76	17	289	870	0.14	0.01	7	23	2	1.5	0.1	0.015	0.001	82	0	1.431	0.091	0.06

[a] Solutions CO_2-sat. (pH 6.3) and illuminated for 15 min with 447 nm illumination with 2.3 W LED at 25 °C with 250 rpm orbital shaking, 1 mL reaction volume. Cat = $CoPyP_{C16}$, PS = $Rubpy_{C17}$, Surf. = $C_{12}E_6$, Red. = NaHAsc, Buf. = phosphate buffer. *Initial pool of experiments. **Experiments from heuristic optimization that were not included in the initial pool. ***Experiments not suggested by the learning algorithm optimization.

Table S8: Selected self-assembled and homogeneous molecular catalyst systems for photocatalytic CO_2-to-CO reduction in aqueous media.

Catalyst (µM)	Photosensitizer (µM)	Reductant (mM)	Environment	Illumination	TOF / min^{-1}	TON	QY / %	Sel_{CO} / %	Ref
$CoPyP_{C16}$ (3.9)	$Rubpy_{C17}$ (112)	NaHAsc (220)	$C_{12}E_6$ micelles (1.425 mM) in 0.462 M H_2PO_4	447 nm 2.3 W LED	25	368	0.15	88	This Work
$CoPyP_{C16}$ (9.8)	$Rubpy_{C17}$ (98)	NaHAsc (348)	$C_{12}E_6$ micelles (1.875 mM) in 0.323 M H_2PO_4	447 nm 2.3 W LED	7.2	184	0.19	87	This Work
$CoPyP_{C16}$ (2.2)	$Rubpy_{C17}$ (109)	NaHAsc (195)	$C_{12}E_6$ micelles (2.4 mM) in 0.509 M H_2PO_4	447 nm 2.3 W LED	28	422	0.10	89	This Work
$CoPyP_{C16}$ (1.5)	$Rubpy_{C17}$ (30)	NaHAsc (100)	$C_{12}E_6$ micelles (0.225 mM) in 0.1 M H_2PO_4	447 nm 2.3 W LED	17	249	0.04	89	This Work
$CoPyP_{C16}$ (0.5)	$Rubpy_{C17}$ (10)	NaHAsc (100)	DMPC, $DSPE^-$ Liposomes in 0.1 M $NaHCO_3$	AM1.5 G 100 mW cm^{-2} λ>400 nm	0.8	189	-	84	[14]
$CoPyP_{C16}$ (0.02)	$Rubpy_{C17}$ (10)	NaHAsc (100)	DMPC, $DSPE^-$ Liposomes in 0.1 M H_2PO_4	AM1.5 G 100 mW cm^{-2} λ>400 nm	6.1	1456	-	77	[14]
$[Re(bpy_{C9})(CO)_3Cl]$ (2.5)	$Rubpy_{C9}$ (2.5)	NaHAsc (100)	DMPC, $DSPE^-$ Liposomes in 0.1 M H_2PO_4	AM1.5 G 100 mW cm^{-2} λ>455 nm	0.08	15	0.05	99	[27]
$[Re(bpy_{C13})(CO)_3Cl]$ (41)	$Rubpy_{C13}$ (40)	NaHAsc (100)	DPPC Liposomes in 0.1 M Tris-HCl (1.0 M)	500 W Xe lamp λ>440 nm	1.1	190	-	>99	[26]
$[Re(bpy_{C13})(CO)_3Cl]$ (41)	-	TEOA (100)	Co-Polymer in H_2O	Xe lamp 106 mW cm^{-2} λ>400 nm	0.02	110	-	>99	[50]
CoTPPS (10)	$[Ru(bpy)_3]Cl_2$ (500)	NaHAsc (100)	Homogeneous in $NaHCO_3$ (0.1 M)	Xe lamp 179 mW cm^{-2} λ>400 nm	3.9	926	-	82	[21]
CoTPPS (0.5)	$[Ru(bpy)_3]Cl_2$ (500)	NaHAsc (100)	Homogeneous in $NaHCO_3$ (0.1 M)	Xe lamp 179 mW cm^{-2} λ>400 nm	16.7	4000	-	41	[21]
CoTCPP (0.25)	$[Ru(bpy)_3]Cl_2$ (500)	NaHAsc (100)	Homogeneous in $NaHCO_3$ (0.1 M)	Xe lamp 179 mW cm^{-2} λ>400 nm	20	2500	0.24	72	[24]
$CoPyP_{Me}$ (5)	Cu Complex (500)	NaHAsc (100)	Homogeneous in $NaHCO_3$ (0.1 M)	Xe lamp 179 mW cm^{-2} λ>400 nm	11.2	2680	1.6	77	[22]
CoTPPS (0.25)	$CuInS_2$-QDs (2.5) with $SC_2H_4NH_3^+$ caps	NaHAsc (25)	Particulate System in $NaHCO_3$ (0.1 M)	LED 140 mW cm^{-2} λ = 450 nm	12.6	72848	0.06	87	[28]

Table S9: Minima and maxima over the training set for Catalyst, Photosensitizer, Surfactant, Reductant, Buffer concentrations.

	[Catalyst] / µM	[Photosensitizer] / µM	[Surfactant] / CMC	[Reductant] / mM	[Buffer] / mM
x_{min}	1.5	10	2.6	1	72
x_{max}	8.6	168	33.7	500	870

Table S10: Coefficients (exponents) for power combinations of the original features.

	$[Catalyst]_{norm}$	$[Photosenzitiser]_{norm}$	$[Surfactant]_{norm}$	$[Reductant]_{norm}$	$[Buffer]_{norm}$
$x_{1,obj1,norm}$	0.216	0.150	0.207	-0.408	-0.850
$x_{2,obj1,norm}$	0.083	0.073	-0.041	-0.509	-0.852
$x_{1,CO,norm}$	-0.421	-0.544	0.323	-0.210	0.606
$x_{2,CO,norm}$	-0.465	-0.499	0.517	-0.487	0.174
$x_{1,TON_{CO},norm}$	0.594	0.393	0.017	0.052	0.700
$x_{2,TON_{CO},norm}$	0.641	0.396	0.215	-0.078	0.616
$x_{1,TOF_{CO},norm}$	-0.127	-0.353	0.371	-0.450	-0.720
$x_{2,TOF_{CO},norm}$	-0.583	-0.583	0.103	-0.424	-0.360
$x_{1,QY,norm}$	-0.722	-0.297	0.094	0.139	0.602
$x_{2,QY,norm}$	-0.627	-0.474	0.143	0.161	0.579

Table S11: Minima and maxima over the training set of the non-normalized optimized mixed features.

	x_{min}	x_{max}
$x_{1,obj1}$	0.587	0.998
$x_{2,obj1}$	0.462	0.979
$x_{1,CO}$	0.583	1.190
$x_{2,CO}$	0.503	1.068
$x_{1,TON_{CO}}$	1.034	2.785
$x_{2,TON_{CO}}$	1.010	2.846
$x_{1,TOF_{CO}}$	0.407	1.001
$x_{2,TOF_{CO}}$	0.329	0.923
$x_{1,QY}$	0.691	1.310
$x_{2,QY}$	0.678	1.239

Supplementary Figures

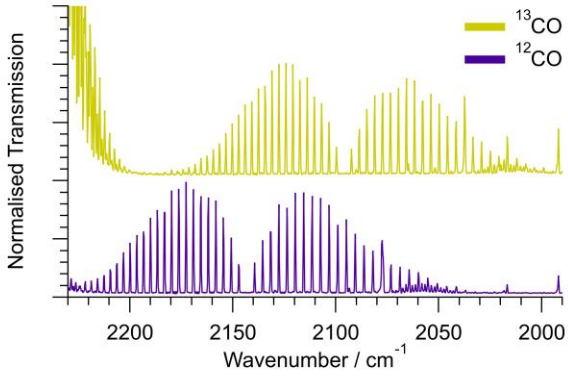

Fig S1: $^{13}CO_2$ isotopic labelling results from reaction mixture of 1.5 µM $CoPyP_{C16}$, 30 µM $Rubpy_{C17}$, 225 µM $C_{12}E_6$ (~3 CMC), 100 mM NaHAsc in CO_2-sat. phosphate buffer (0.1 M, pH 6.3) after 447 nm illumination with 2.3 W LED at 25 °C with 250 rpm orbital shaking.

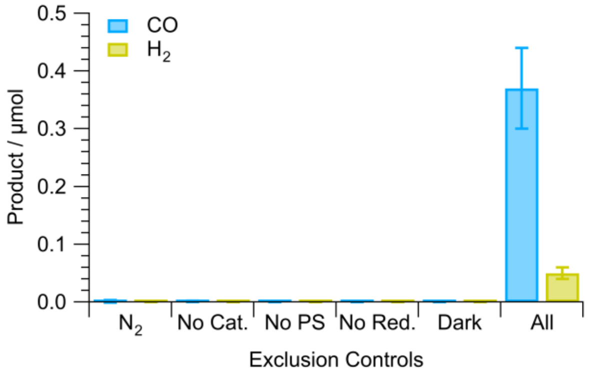

Fig S2: Photocatalytic system exclusion controls. Unless specified, solution of 1.5 µM $CoPyP_{C16}$, 30 µM $Rubpy_{C17}$, 225 µM $C_{12}E_6$ (~3 CMC), 100 mM NaHAsc, phosphate (0.1 M), CO_2-sat. (pH 6.3) after 15 min 447 nm illumination with 2.3 W LED at 25 °C with 250 rpm orbital shaking, 1 mL reaction volume.

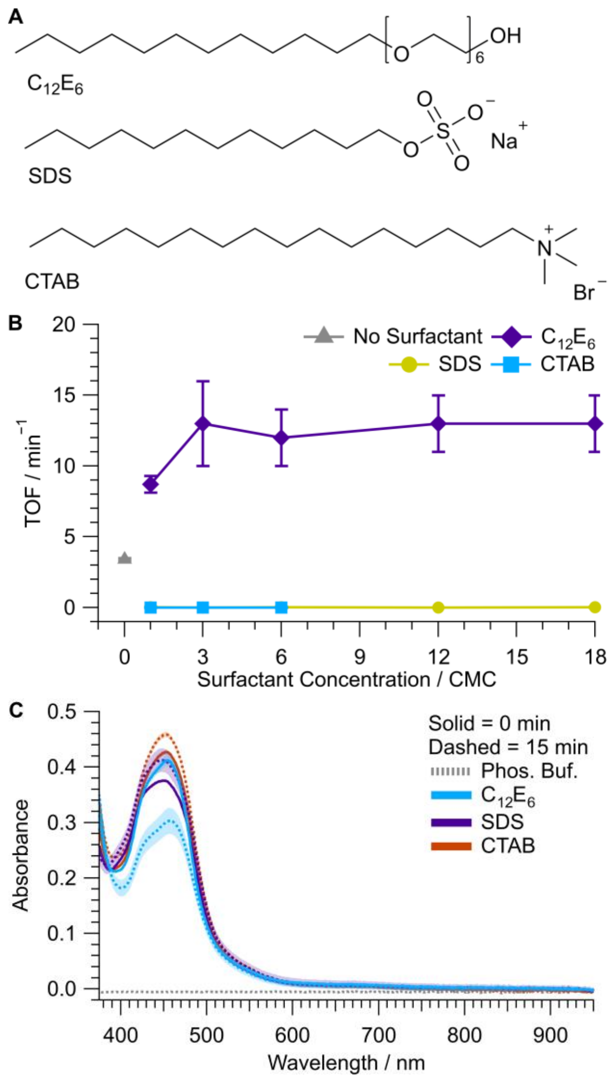

Fig S3: Chemical structures of non-ionic surfactant C12E6, anionic surfactant SDS and cationic surfactant CTAB (A); comparison of photocatalytic performance when using no additional surfactant, non-ionic surfactant C12E6, anionic surfactant SDS and cationic surfactant CTAB (B); and UV-Vis spectra averaging all surfactant concentrations for each surfactant before and after photocatalytic testing (C). Note that time 0 min UV-Vis spectra were measured before CO2 purging, meaning evaporation during purging leads to a minor concentration increase. Surfactant concentration of 75 µM × CMC for C12E6, 7.8 mM × CMC for SDS and 1.0 mM × CMC for CTAB. Solution of 1.5 µM $CoPyP_{C16}$, 30 µM $Rubpy_{C17}$, 100 mM NaHAsc, phosphate (0.1 M), CO_2-sat. (pH 6.3). Photocatalysis test: 15 min 447 nm illumination with 2.3 W LED at 25 °C with 250 rpm orbital shaking, 1 mL reaction volume.

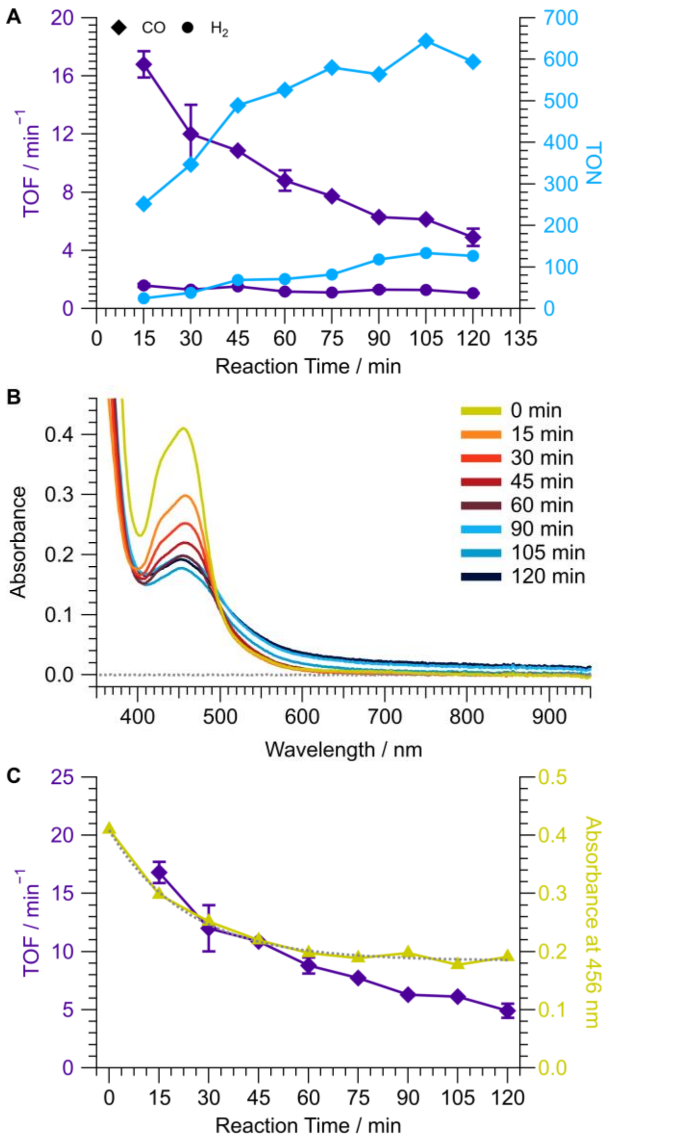

Fig S4: Catalyst turnover frequency (TOF) and turnover number (TON) for CO and H_2 production over time (A); UV-Vis spectra pre/post-reaction (B) and TOF and absorbance at 456 nm, with exponential fit (τ = 23±2 min), over time (C). Reaction with micellar solution of 1.5 µM $CoPyP_{C16}$, 30 µM $Rubpy_{C17}$, 225 µM $C_{12}E_6$ (~3 CMC), 100 mM NaHAsc in CO_2-sat. phosphate buffer (0.1 M, pH 6.3) after 447 nm illumination with 2.3 W LED at 25 °C with 250 rpm orbital shaking, 1 mL reaction volume.

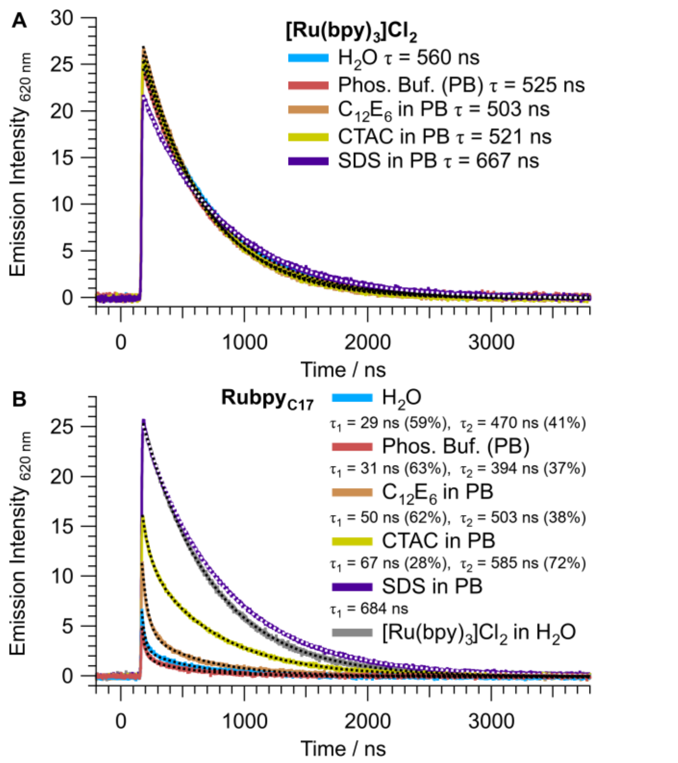

Fig S5: Photoluminescence decay traces (pump 460 nm, probe 620 nm) of Rubpy (A) and $Rubpy_{C17}$ (B) absent ascorbate quencher. 30 µM photosensitizer in Ar purged aqueous media; phosphate buffer (0.1 M, pH 7.0) and/or surfactant (3 CMC) present as indicated.

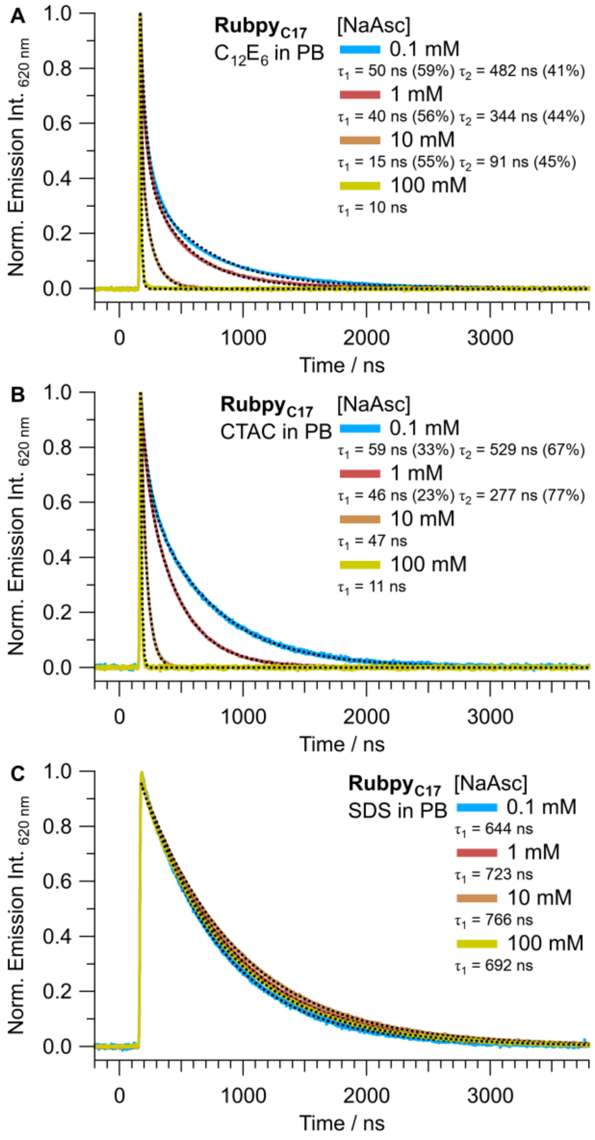

Fig S6: Photoluminescence decay traces (pump 460 nm, probe 620 nm) of Rubpy$_{C17}$ with varying ascorbate quencher concentration in $C_{12}E_6$ (A), CTAC (B) and SDS (C) micelles. 30 µM photosensitizer in phosphate buffer (0.1 M, pH 7.0), Ar purged; surfactant at 3 CMC.

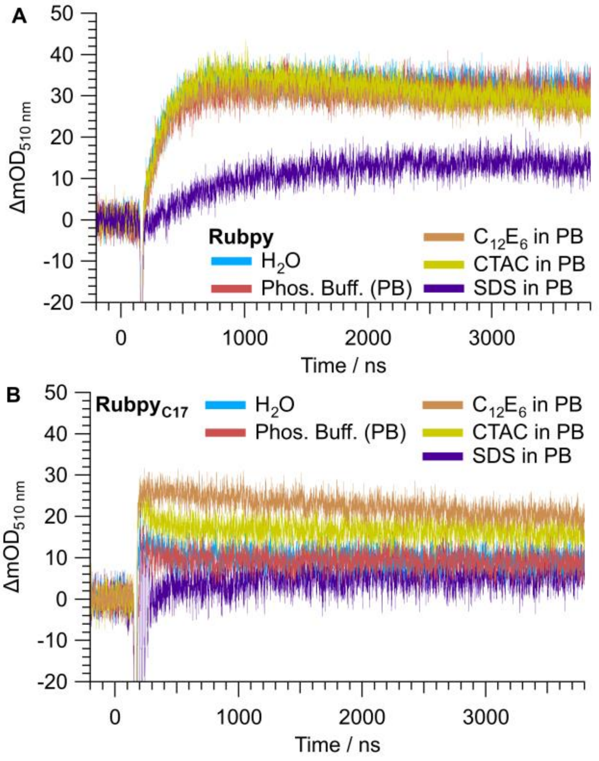

Fig S7: Transient absorption spectroscopy (pump 460 nm, probe 510 nm) of Rubpy (A) and Rubpy$_{C17}$ (B). 30 µM photosensitizer, 100 mM sodium ascorbate in phosphate buffer (0.1 M, pH 7.0), Ar purged; surfactant at 3 CMC if present.

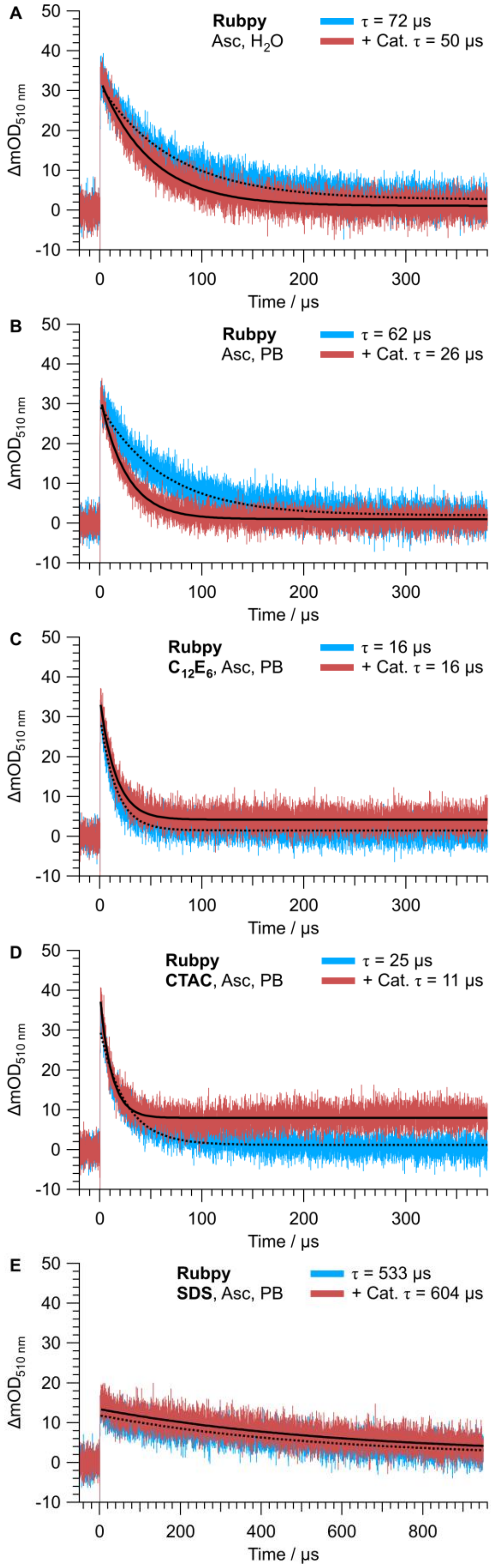

Fig S8: Transient absorption spectroscopy (pump 460 nm, probe 510 nm) of Rubpy with and without 1.5 µM CoPyP$_{C16}$. 30 µM photosensitizer, 3 CMC of surfactant (where specified), 100 mM sodium ascorbate in phosphate buffered (0.1 M, pH 7.0) Ar purged solution.

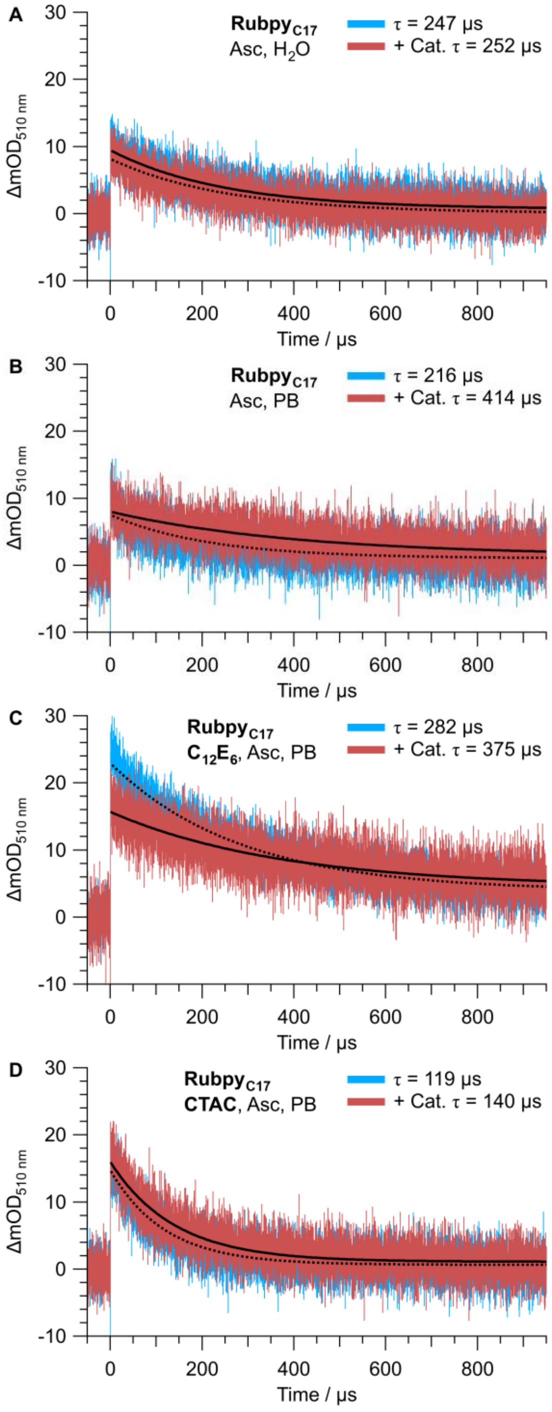

Fig S9: Transient absorption spectroscopy (pump 460 nm, probe 510 nm) of Rubpy$_{C17}$ with and without 1.5 µM CoPyP$_{C16}$. 30 µM photosensitizer, 3 CMC of surfactant (where specified), 100 mM sodium ascorbate in phosphate buffered (0.1 M, pH 7.0) Ar purged solution.

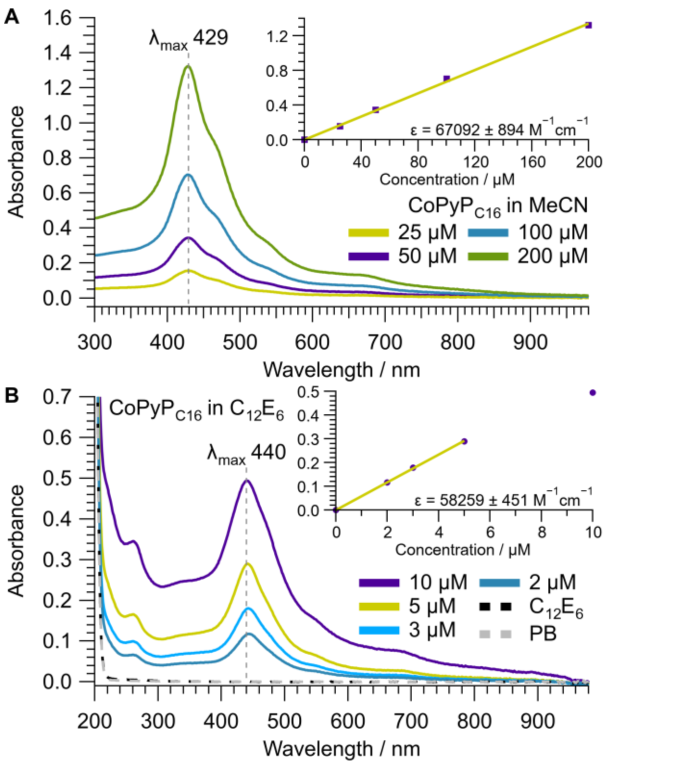

Fig S10: Molar extinction coefficient determination for $CoPyP_{C16}$ showing UV-Vis spectra in (A) MeCN and (B) $C_{12}E_6$ (225 µM, ~3 CMC) in H_2O, with linear fitting of absorption maxima inset. Both fittings exhibit a correlation coefficient (r^2) of 1 with 1% error in the fitting.

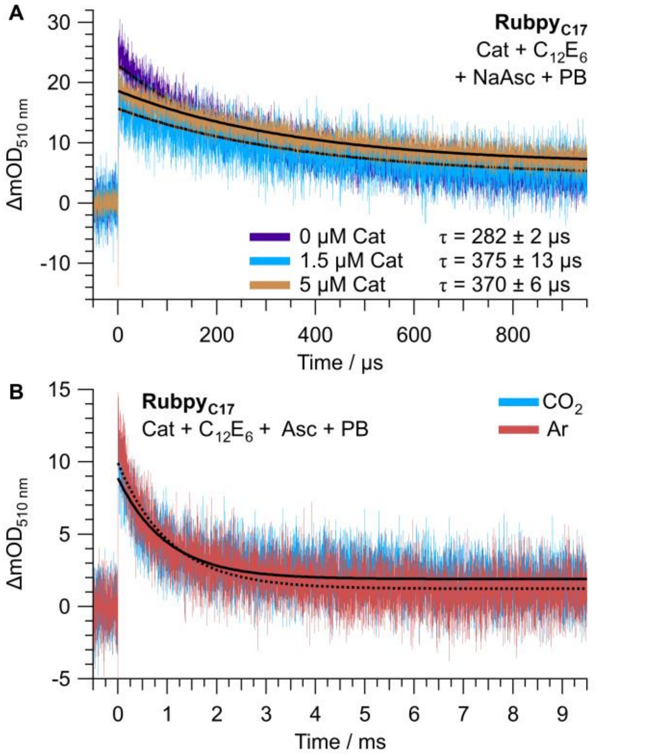

Fig S11: Transient absorption spectroscopy (pump 460 nm, probe 510 nm) of $Rubpy_{C17}$ with differing concentrations of $CoPyP_{C16}$ in phosphate buffered (0.1 M, pH 7.0) Ar purged solution (A); and 5µM $CoPyP_{C16}$ in Ar (pH 7.0) and CO_2 (pH 6.3) saturated phosphate buffered (0.1 M) solution. 30 µM photosensitizer, 3 CMC $C_{12}E_6$, 100 mM sodium ascorbate

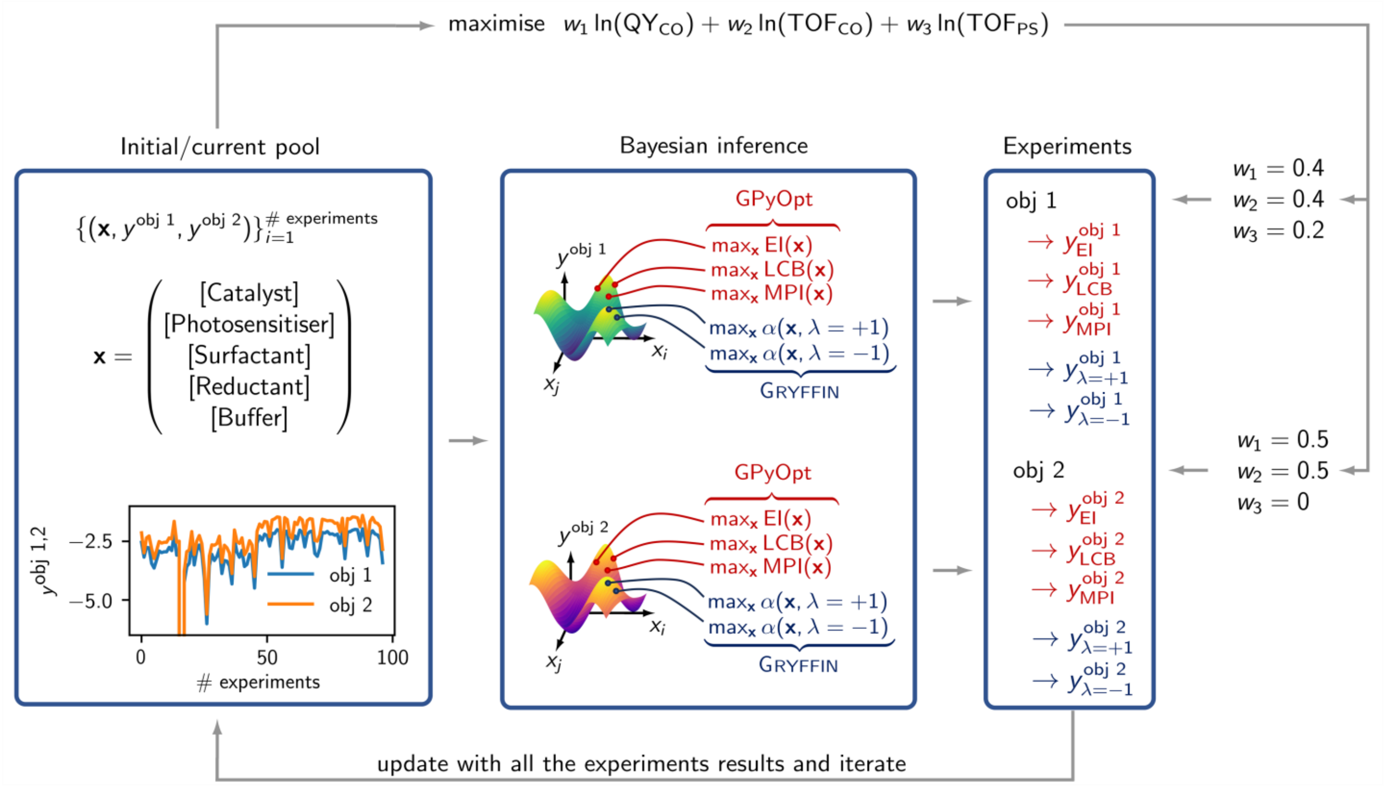

Fig S12: Overview of the protocol used for the orchestration of the photocatalytic micelles experiments towards the simultaneous optimisation of two objective functions. An initial pool of already performed experiments is considered, with **x** being the features array, $y^{obj\ 1}$ the respective responses for objective function 1, $y^{obj\ 2}$ the respective responses for objective function 2. Two methodologies, GPyOpt and Gryffin, are employed; for getting the next features combination to be tested, the former allows the use of three acquisition functions (EI, LCB, MPI), the latter of two acquisition functions ($\lambda = \pm1$). The resulting 10 features arrays are experimented, and, together with the corresponding responses, are used to update the initial pool for the next iteration.

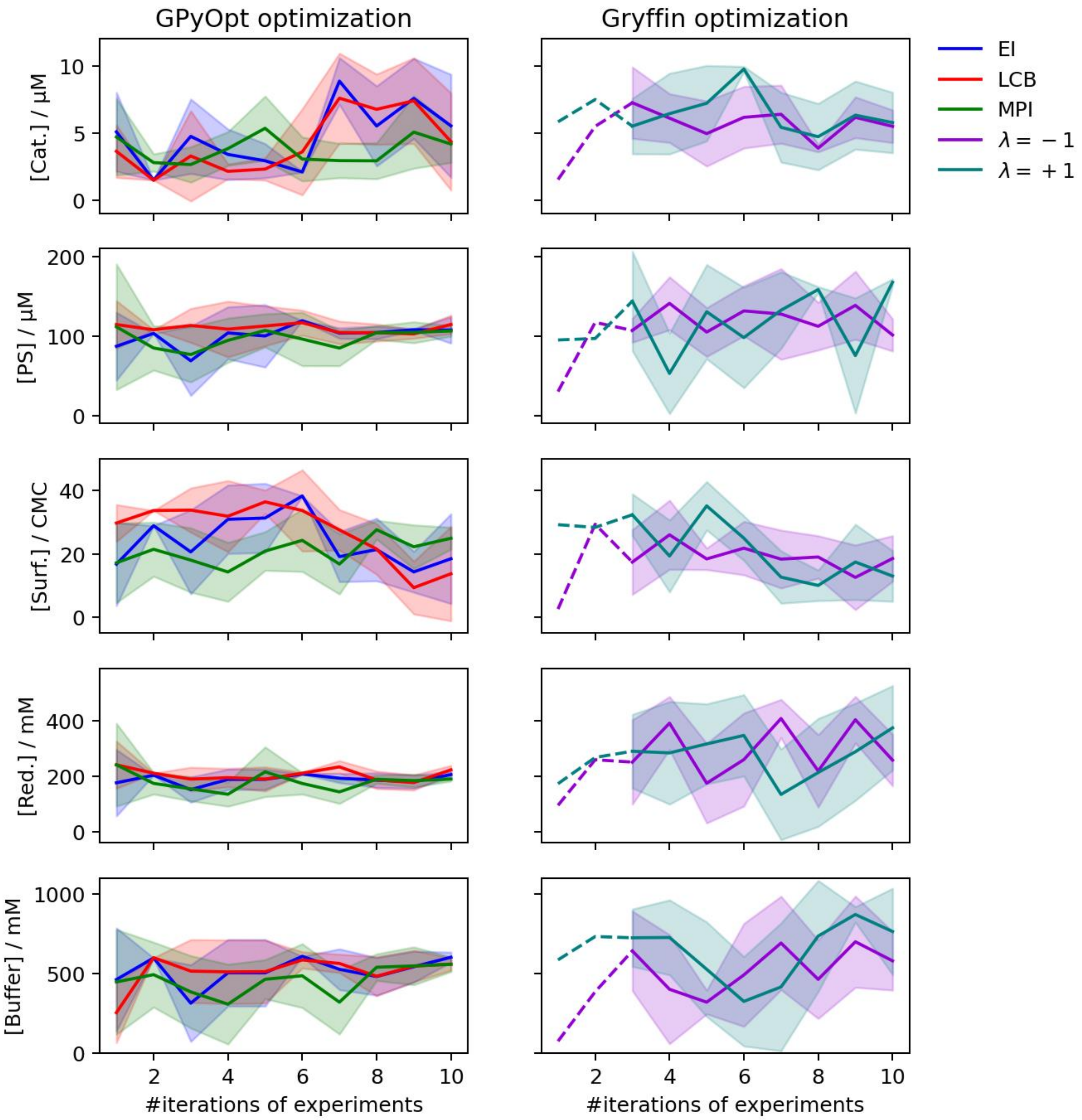

Fig S13: Predicted concentrations (mean±standard deviation) of [Catalyst], [Photosensitizer], [Surfactant], [Reductant], [Buffer] from 5 replicate Bayesian Optimization algorithm runs for Obj 1, over the 10 experimental iterations for GPyOpt, with acquisition functions EI, LCB, MPI; and for Gryffin, with acquisition functions given by λ=-1 (explorative) and λ=+1 (exploitative). Dashed values indicate means for which there is no available standard deviation.

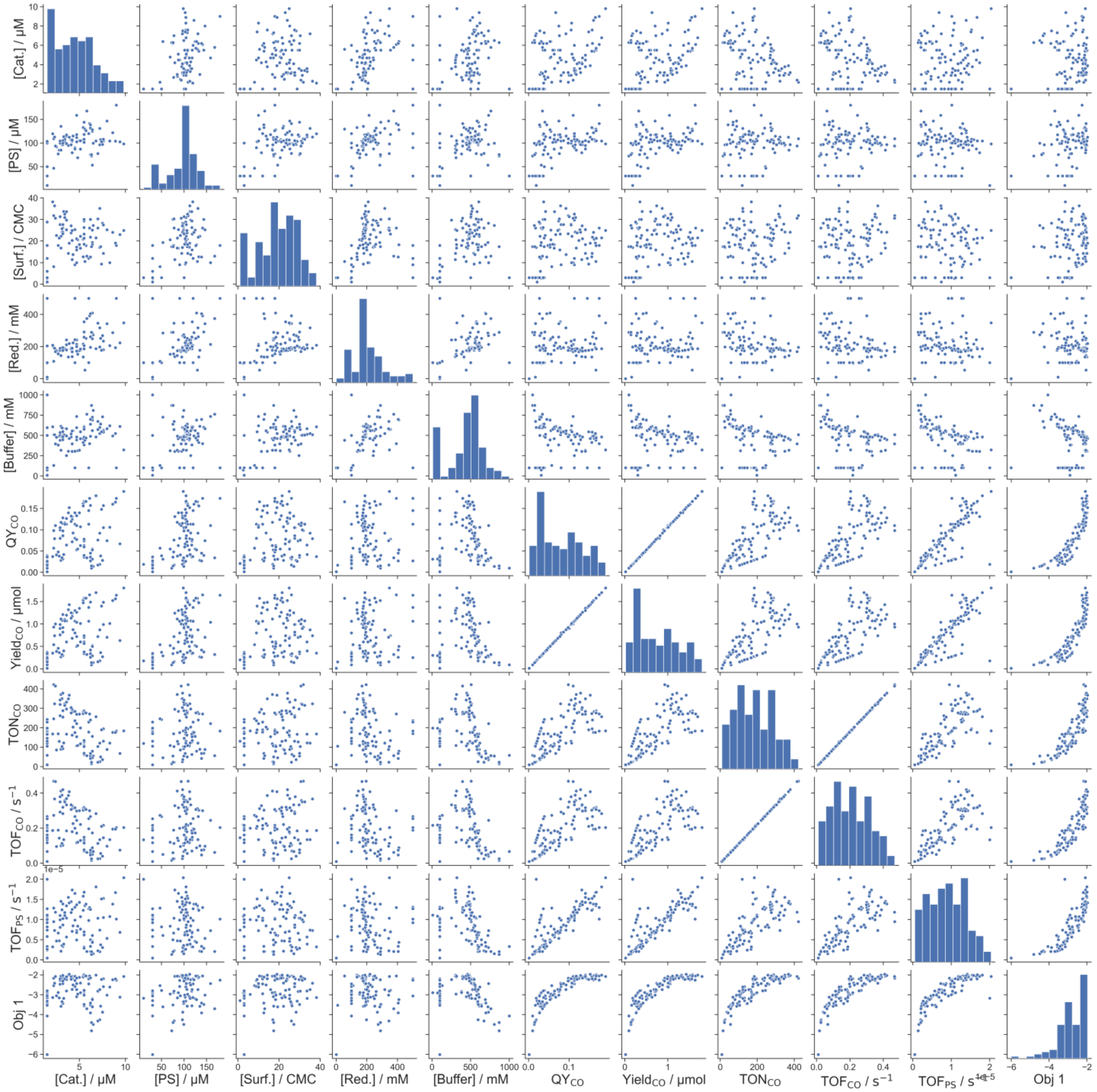

Fig S14: Correlation pair-plot matrix for all the possible couples of parameters and performance metrics for all the 103 experimentally tested combinations. Clear linear correlations are observed between TOF and TON as well as Yield and QY as one is the other divided by a constant, respectively. The correlation coefficients for each plot are shown in a matrix in Fig S15.

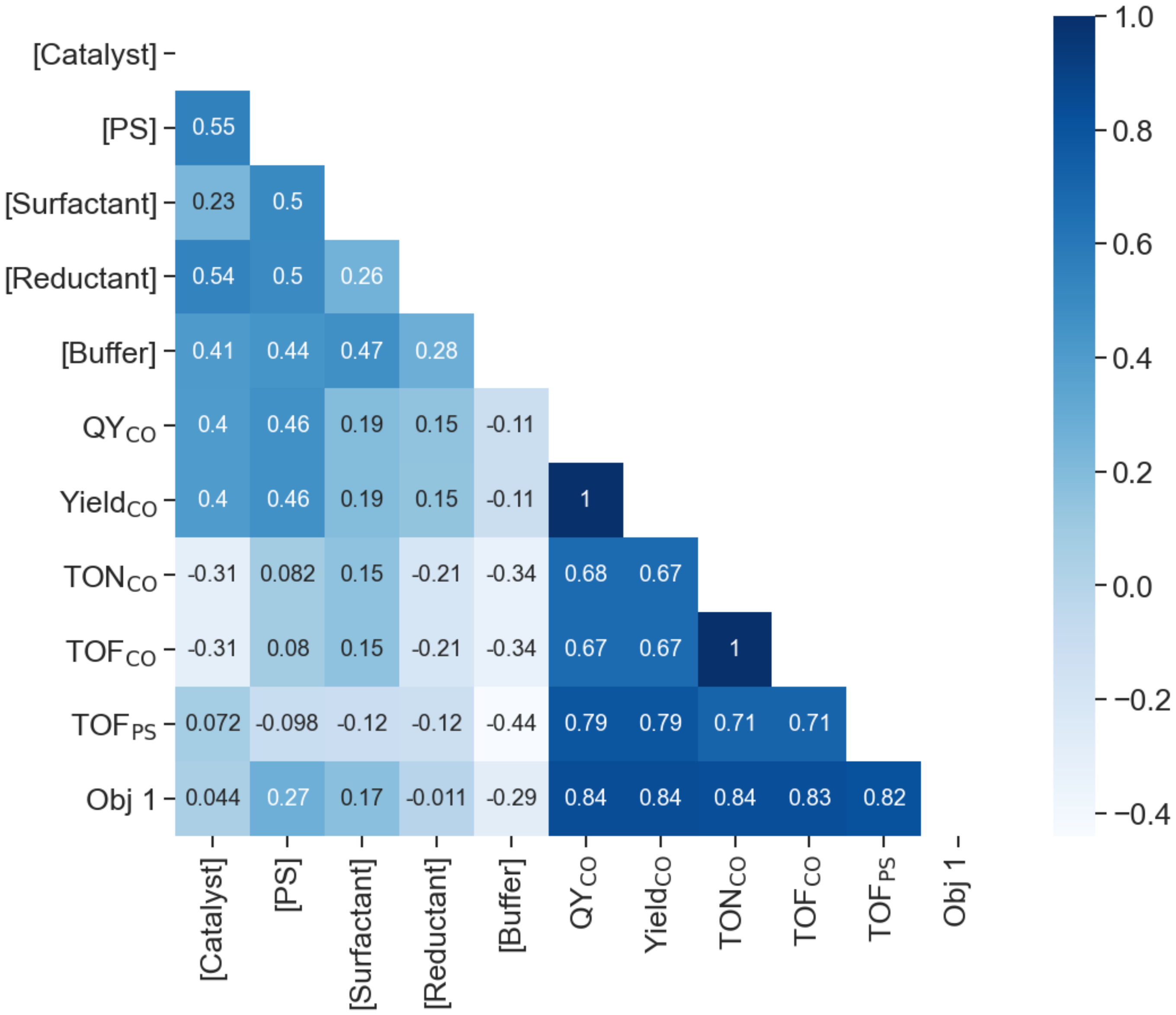

Fig S15: Correlation matrix with linear correlation coefficients for all the possible couples of both parameters and performance metrics calculated over the 103 experimentally tested combinations. Clear linear correlations are observed between TOF and TON as well as Yield and QY as one is the other divided by a constant, respectively.

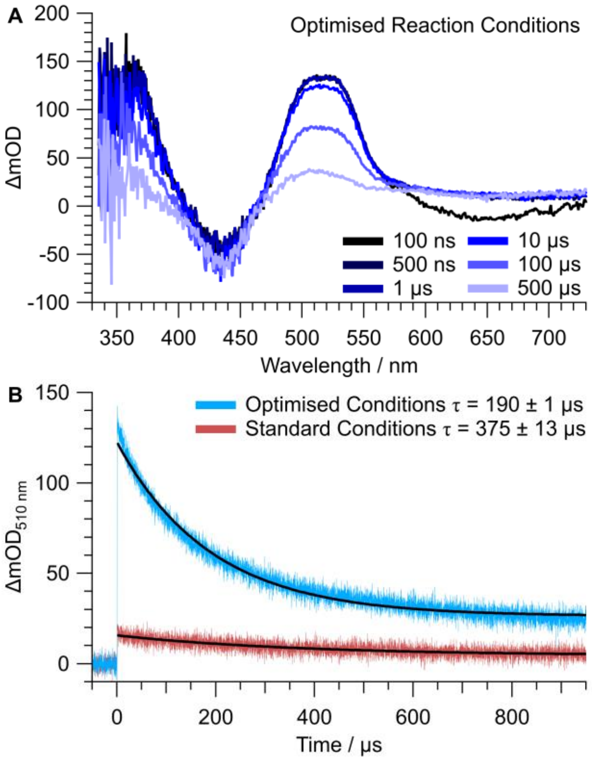

Fig S16: Transient absorption spectra of algorithm optimized photocatalytic system (A); kinetic traces (pump 460 nm, probe 510 nm) of optimized and starting conditions (B). Optimized conditions: 3.9 µM CoPyP$_{C16}$, 112 µM Rubpy$_{C17}$, 1425 µM / 19 CMC $C_{12}E_6$, 220 mM sodium ascorbate in 462 mM phosphate buffer (pH 7.0), Ar purged; Standard conditions: 1.5 µM CoPyP$_{C16}$, 30 µM Rubpy$_{C17}$, 225 µM / 3 CMC $C_{12}E_6$, 100 mM sodium ascorbate in 100 mM phosphate buffered (pH 7.0), Ar purged.

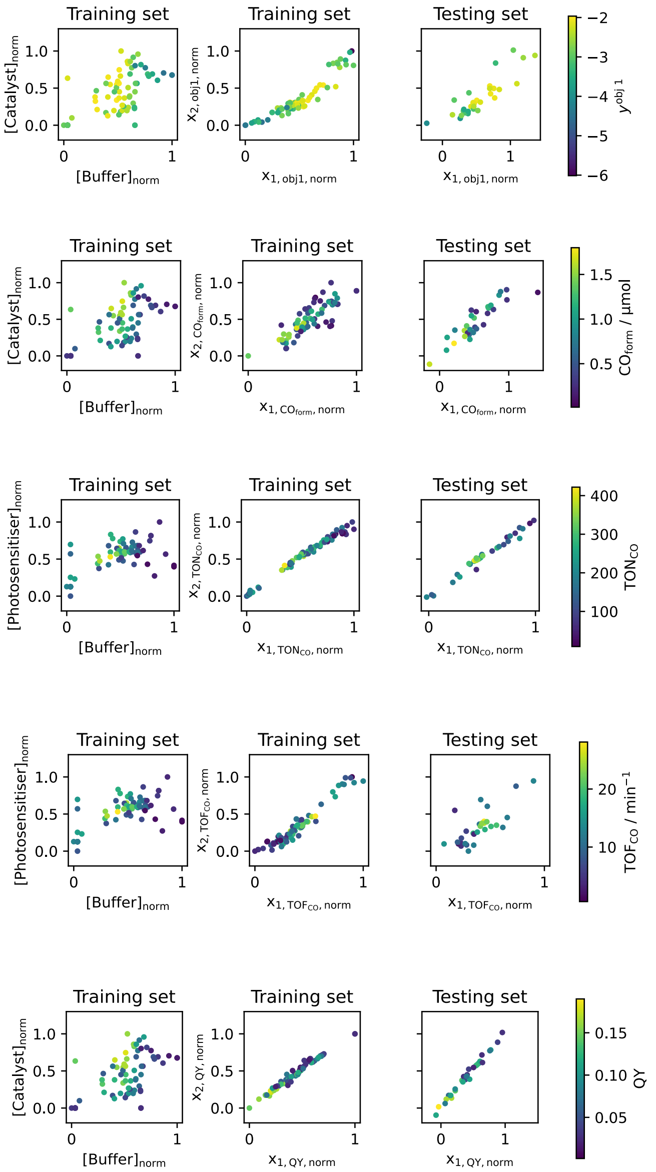

Fig S17: Control Group Feature analysis comparing training sets using the top two features according to the SHAP ranking and using the 'optimized features' that are obtained as power combinations of the catalyst, photosensitizer, surfactant, reductant and buffer concentrations; and exemplifying the predictive power with the testing set. Results are reported for **a** $y^{obj\,1}$, **b** CO_{form}, **c** TON_{CO}, **d** TOF_{CO}, **e** QY. When plotted against mixed features, $y^{obj\,1}$ shows optimal values for $x_{1,obj1,norm}$ and $x_{2,obj2,norm}$ about 0.8 and 0.5 respectively; CO_{form} shows optimal values for $x_{1,COform,norm}$ and $x_{2,COform}$,norm about 0.3; QY shows optimal values for $x_{1,QY,norm}$ and $x_{2,QY,norm}$ below 0.2. No clear indication appears for TON_{CO} and TOF_{CO}.
